# CTIP2-Regulated Reduction in PKA-Dependent DARPP32 Phosphorylation in Human Medium Spiny Neurons: Implications for Huntington Disease

**DOI:** 10.1016/j.stemcr.2019.07.015

**Published:** 2019-08-22

**Authors:** Marija Fjodorova, Morgane Louessard, Zongze Li, Daniel C. De La Fuente, Emma Dyke, Simon P. Brooks, Anselme L. Perrier, Meng Li

**Affiliations:** 1 Neuroscience and Mental Health Research Institute, School of Medicine, Cardiff University, Cardiff CF24 4HQ, UK; 2 Division of Neuroscience, School of Bioscience, Cardiff University, Cardiff CF10 3AX, UK; 3 Institut National de la Santé et de la Recherche Médicale (INSERM) UMR861, I-Stem, AFM, 91100 Corbeil-Essonnes, France

**Keywords:** CTIP2, DARPP32, Huntington disease, medium spiny neuron, neural differentiation

## Abstract

The mechanisms underlying the selective degeneration of medium spiny neurons (MSNs) in Huntington disease (HD) remain largely unknown. CTIP2, a transcription factor expressed by all MSNs, is implicated in HD pathogenesis because of its interactions with mutant huntingtin. Here, we report a key role for CTIP2 in protein phosphorylation via governing protein kinase A (PKA) signaling in human striatal neurons. Transcriptomic analysis of CTIP2-deficient MSNs implicates CTIP2 target genes at the heart of cAMP-Ca^2+^ signal integration in the PKA pathway. These findings are further supported by experimental evidence of a substantial reduction in phosphorylation of DARPP32 and GLUR1, two PKA targets in CTIP2-deficient MSNs. Moreover, we show that CTIP2-dependent dysregulation of protein phosphorylation is shared by HD hPSC-derived MSNs and striatal tissues of two HD mouse models. This study therefore establishes an essential role for CTIP2 in human MSN homeostasis and provides mechanistic and potential therapeutic insight into striatal neurodegeneration.

## Highlights

• CTIP2 protects human MSNs against oxidative stress-induced cell death
• PKA-regulated DARPP32 and GLUR1 phosphorylation is reduced in CTIP2-deficient MSNs
• HD MSNs share CTIP2-dependent downregulation of protein phosphorylation by PKA

## Teaser

In this article, Fjodorova and colleagues provide mechanistic and potential therapeutic insight into striatal neurodegeneration caused by CTIP2 hypofunction. CTIP2 plays an essential role in human MSN homeostasis via regulating PKA-dependent DARPP32 phosphorylation and protecting MSNs from oxidative stress-induced cell death. Protein phosphorylation deficits occur in human and mouse Huntington disease MSNs due to CTIP2- and mHTT-co-regulated molecular signaling abnormalities.

## Introduction

Inhibitory γ-amino butyric acid (GABA)-releasing medium spiny neurons (MSNs) are the principal projection neurons of the basal ganglia, receiving inputs from both glutamatergic cortical neurons and midbrain dopaminergic neurons. DARPP32 is a class-defining protein marker for striatal MSNs and a central mediator of dopaminergic and other first-messenger signaling in these cells. Specifically, phosphorylation of DARPP32 at threonine 34 (pDARPP32-Thr34) by protein kinase A (PKA), following dopamine D1 receptor activation, is critically involved in regulating electrophysiological, transcriptional and behavioral responses of MSNs to physiological and pharmacological stimuli, including antidepressants, neuroleptics, and drugs of abuse (Yger and Girault, 2011). Thus, these findings point to an important role for PKA-regulated phosphorylation of DARPP32 in MSN health, with major implications for neurological disorders.

CTIP2 (also known as BCL11B) is a transcription factor expressed by all MSNs and is required for MSN development and transcriptional regulation of striatal genes (Arlotta et al., 2008, Onorati et al., 2014). CTIP2 deficiency results in structural striatal defects, impaired spatial learning, and working memory deficits in mice (Arlotta et al., 2008, Simon et al., 2016). Furthermore, CTIP2 protein levels are reduced in both human and rodent mutant huntingtin (mHTT)-expressing cells before the onset of MSN degeneration (Langfelder et al., 2016, Ring et al., 2015), pointing to a role for CTIP2 in Huntington disease (HD) pathogenesis. These findings have led us to hypothesize that CTIP2 may play an important role in conferring regional specificity of HD neurodegeneration that cannot be explained by the ubiquitous expression of the mHTT. However, whether CTIP2 deficiency directly leads to increased MSN vulnerability and dysfunction remains to be demonstrated.

Using CRISPR/Cas9 genome-edited human embryonic stem cells (hESCs) as a model, we uncover a role for CTIP2 in regulating protein kinase and phosphatase levels and activity and subsequently phosphorylation of DARPP32-Thr34 in MSNs. We show for the first time that deficits in PKA-dependent protein phosphorylation occur in human and mouse HD MSNs, potentially owing to CTIP2- and mHTT-co-regulated molecular signaling abnormalities as suggested by transcriptomic analysis. This study provides evidence of a central role for CTIP2 in human MSN homeostasis and supports the hypothesis that CTIP2 may mediate regional specificity of HD pathogenesis.

## Results

### CTIP2 Deficiency Does Not Compromise the Generation of MSNs from hESCs

**Figure 1:**
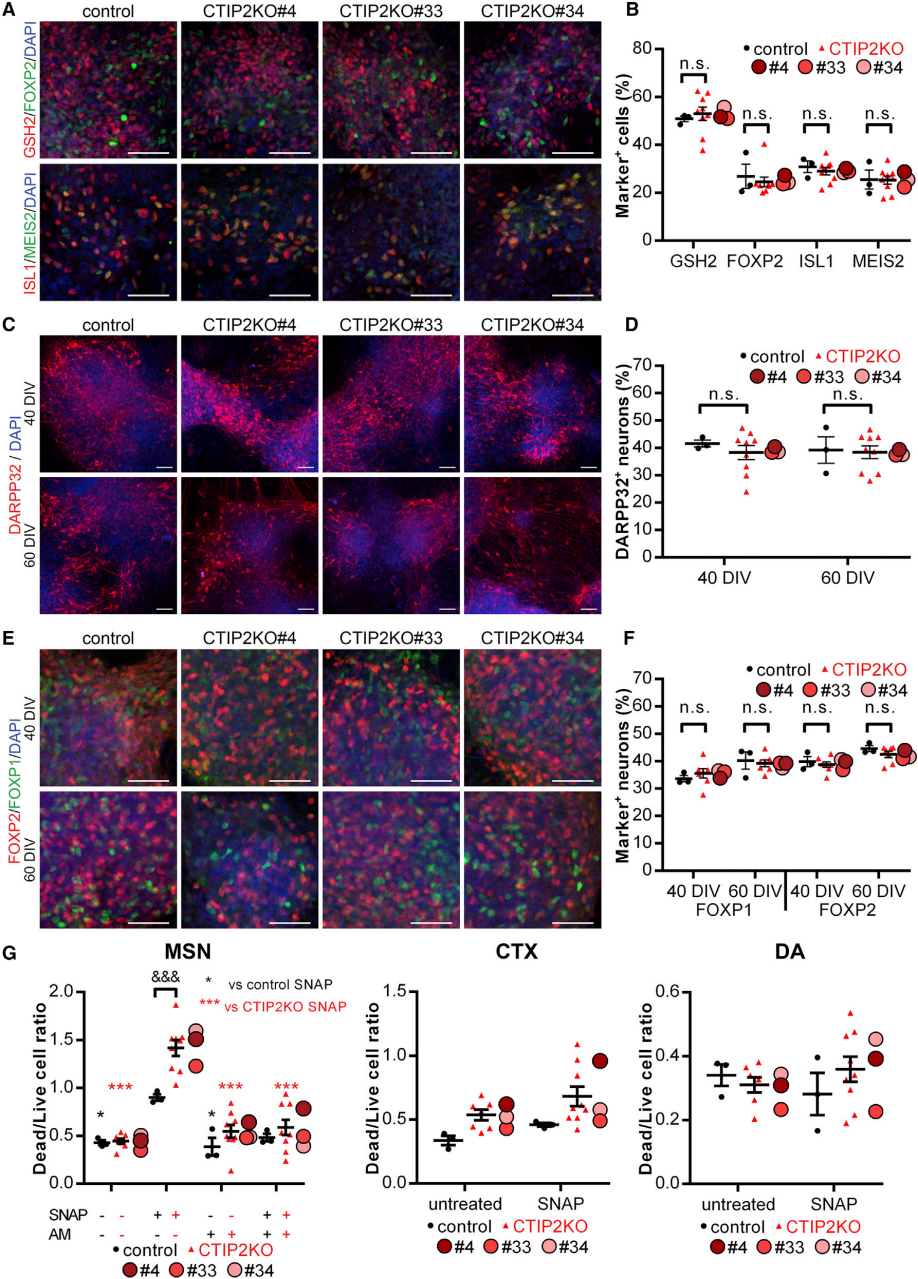
CTIP2KO MSNs Acquire Normal Striatal Cell Identity but Present with Increased Vulnerability to Oxidative Stress (A and B) LGE-like progenitors in control and CTIP2KO cultures at 20 DIV labeled and quantified for GSH2, FOXP2, ISL1, and MEIS2 (n = 3, 9, 3). (C and D) MSNs in control and CTIP2KO cultures at 40 and 60 DIV labeled and quantified for DARPP32 (n = 3, 9, 3). (E and F) MSNs in control and CTIP2KO cultures at 40 and 60 DIV labeled and quantified for FOXP1 and FOXP2 (n = 3, 7, ≥2). (G) Pre-treatment of MSNs with 50 μM amentoflavone (AF) for 2 h protects them from SNAP-induced cell death at 40 DIV. Similar vulnerability to oxidative stress is observed between control and CTIP2-deficient groups in both cortical neurons (CTX) and dopaminergic (DA) neurons (n = 3, 9, 3). (A, C, and E) Scale bars, 50 μm. (B, D, and F) One-way ANOVA; n.s., not significant. (G) Two-way ANOVA; MSN: ^∗^p < 0.05, ^∗∗∗^p < 0.001; ^&&&^p < 0.001. Data are presented as mean ± SEM for each genotype, with the means for individual clones indicated by red-shaded circles beside CTIP2KO data. See also Figures S1 and S2.

Homozygous CTIP2 knockout (KO) hESC lines were generated using the CRISPR/Cas9-assisted genome editing technology with guide RNAs targeting exon 2 of the *CTIP2* gene (Figures S1A–S1D). To investigate whether CTIP2 deficiency affects the generation of MSNs from hESCs, we performed striatal neural differentiation on CTIP2KO and control lines using an established protocol (Arber et al., 2015). At 20 days *in vitro* (DIV), both control and CTIP2KO hESCs gave rise to high yields of cells expressing lateral ganglionic eminence (LGE) markers GSX2, FOXP2, ISL1, and MEIS2, subpallial marker ASCL1, with very few TBR1^+^ cortical cells (Figures 1A, 1B, and S2). These LGE-like progenitors differentiated into 35%–40% DARPP32^+^, FOXP1^+^, and FOXP2^+^ MSNs by 40 DIV and maintained MSN identity for the subsequent 20-day period (Figures 1C–1F). Taken together, these results suggest that the loss of CTIP2 does not affect the yield of nascent and mature MSNs derived from hESCs.

### CTIP2-Deficient MSNs Display Increased Vulnerability to Oxidative Stress

We then asked whether loss of CTIP2 would compromise MSN health. To this extent we investigated oxidative stress-dependent cell death using the nitric oxide donor *S*-nitroso-*N*-acetylpenicillamine (SNAP), which was previously shown to generate reactive oxygen species and induce oxidative stress (Wei et al., 2000). After a 24-h exposure of cells to SNAP we observed a 2-fold greater increase in apoptosis in CTIP2KO MSNs compared with control neurons (Figure 1G). This phenotype was rescued by a 2-h pre-treatment with the neuroprotective and anti-apoptotic agent amentoflavone (Figure 1G, MSN), a drug previously shown to reduce neuronal damage via decreasing nitric oxide production in response to apoptotic stimuli (Zhang et al., 2015). We next checked if CTIP2-dependent cell death deficits were MSN specific by analyzing differentiated CTIP2-expressing cortical neurons and dopamine neurons that do not express CTIP2. Interestingly, cortical neurons were less vulnerable to oxidative stress than MSNs, with CTIP2KO neurons showing only a trend toward a more severe phenotype than control (Figure 1G, CTX). Oxidative stress-dependent cell death levels were not affected by CTIP2KO in dopaminergic neurons (Figure 1G, DA). This data suggests that CTIP2 plays a neuroprotective role against oxidative stress that is restricted to MSNs.

### Genome-wide Transcriptome Analysis Highlights a Role for CTIP2 in PKA-Regulated Protein Phosphorylation

To investigate the molecular mechanisms and pathways leading to pathological changes in CTIP2-deficient MSNs, we performed whole-transcriptome RNA sequencing (RNA-seq) analysis of CTIP2KO no. 4 and control MSN cultures at 20 and 40 DIV (MSN20 and MSN40, respectively). Global principal component analysis showed clear sample segregation based on genotype and developmental stage (Figure S3A). Analysis of protein-coding genes identified 4,903 and 5,835 differentially expressed genes (DEGs) for MSN20 and MSN40, respectively (Figure S3B; Table S1). Moreover, MSN20 and MSN40 DEGs were enriched for human striatum-specific genes and significantly associated with the Ctip2 loss-of-function study in mouse striatum (Arlotta et al., 2008, Onorati et al., 2014) (Table S2). Among these striatal genes are *EBF1*, *LMO3*, *CNR1*, *DRD1*, *DRD2*, *SST*, *REC8*, *GRM1*, and *PDE5A*, which were all downregulated in CTIP2KO cells.

**Figure 2:**
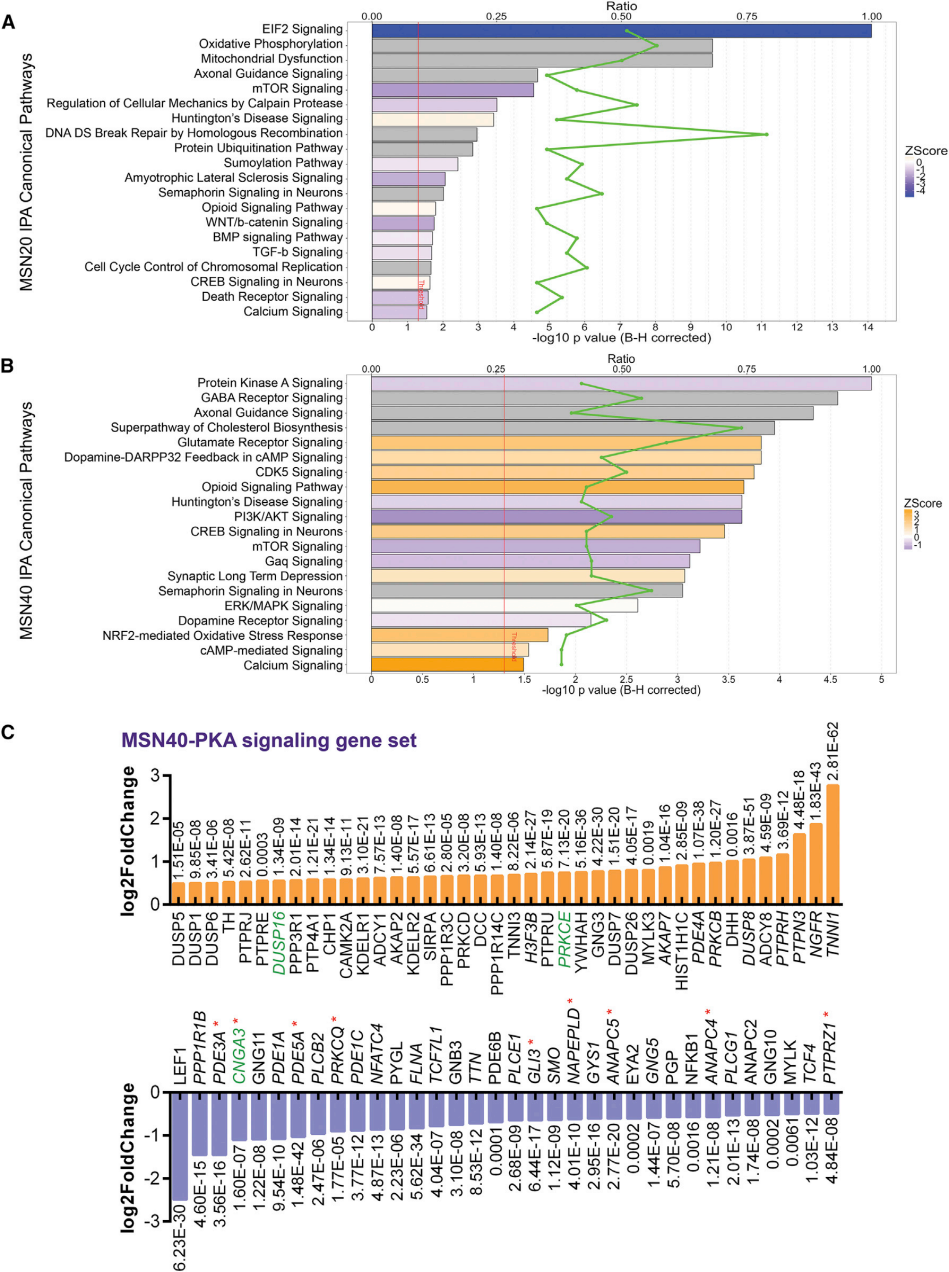
RNA-Seq Analysis Highlights a Role for CTIP2 in PKA-Regulated Protein Phosphorylation in hESC-Derived MSNs (A and B) Ingenuity pathway analysis (IPA) of MSN20 (A) and MSN40 (B) CTIP2KO DEGs shows significant enrichment of genes regulating mitochondrial function at 20 DIV, PKA and CDK5 signaling as well as dopamine-DARPP32 feedback in cAMP signaling at 40 DIV (full gene set lists for both time points are presented in Table S2). (C) Differential expression statistics for MSN40 DEGs within the PKA gene set at fold change threshold of |1.4|, with direct CTIP2 targets highlighted in green. Genes analyzed by RT-PCR in independent control and CTIP2KO MSN samples are indicated in italic, with red asterisks marking validated differential expression. See also Figure S3 and Tables S2 and S3.

**Figure 3:**
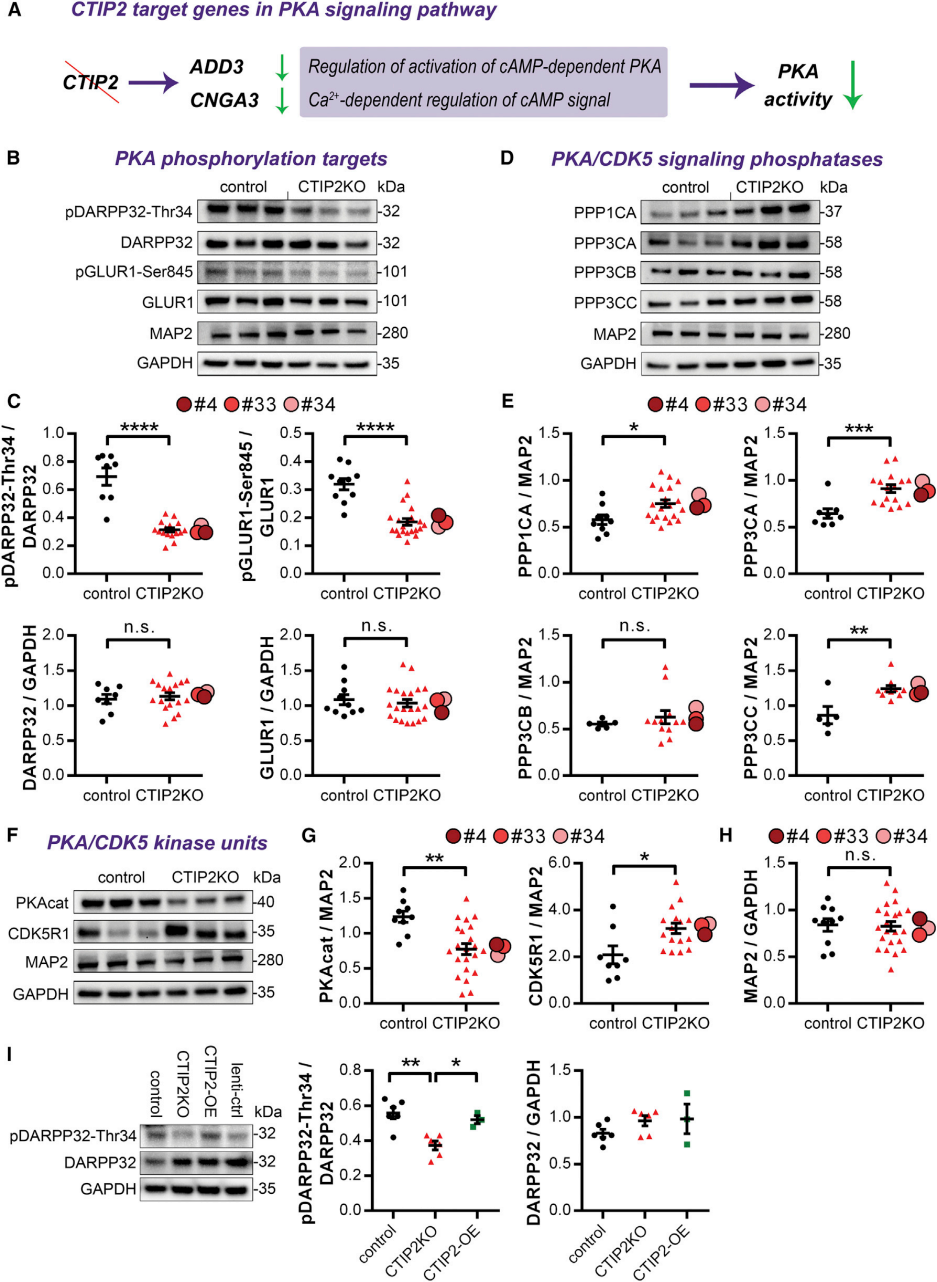
PKA-Dependent DARPP32-Thr34 and GLUR1-Ser845 Phosphorylation Is Regulated by CTIP2 in MSNs (A) Within the PKA signaling gene set, CTIP2 target genes (*ADD3* and *CNGA3*) were significantly dysregulated and their change predicted to inhibit activation of cAMP-dependent PKA. (B) Phosphorylation of PKA targets DARPP32-Thr34 and GLUR1-Ser845 is greatly reduced in CTIP2KO versus control MSNs. (C) Quantification of (B) (left: n = 8, 17, 4; right: n = 10, 21, 4). (D) Part of the PKA signaling pathway, levels of protein phosphatase 1 (PP1) and 3 (PP3) catalytic subunits are significantly increased in CTIP2KO versus control MSNs. (E) Quantification of (D) (from left, top row: n = 9, 20, 4; n = 8, 16, 3; bottom row: n = 5, 12, 3; n = 5, 10, 3). (F) Compared with control cells, CTIP2KO MSNs contain reduced levels of PKA catalytic subunits (PKAcat) and increased levels of CDK5R1, a neuron-specific activator of CDK5. (G) Quantification of (F) (from left: n = 9, 22, 4; n = 8, 16, 3). (H) Quantification of MAP2 levels shows no differences between control and CTIP2KO MSN cultures (n = 10, 22, 4). (I) Reduced phosphorylation of DARPP32-Thr34 in CTIP2KO no. 4 MSNs is rescued by restoring CTIP2 levels (n = 6, 6, 3). (C, E, and G–I) One-way ANOVA; ^∗^p < 0.05, ^∗∗^p < 0.01, ^∗∗∗^p < 0.001, ^∗∗∗∗^p < 0.0001; n.s., not significant. Data are presented as mean ± SEM for each genotype, with the means for individual clones indicated by red-shaded circles beside CTIP2KO data. See also Figures S1E and S4.

We identified mitochondrial dysfunction, oxidative phosphorylation, calcium signaling, and HD signaling among the significantly dysregulated pathways at both time points (Figures 2A, 2B, and S3C; Table S2). Intriguingly, genes concerning dopamine-DARPP32 feedback in cAMP signaling, PKA and CDK5 signaling were found to be significantly altered in mature MSNs (Figure 2B). Also, significant dysregulation of synaptic signaling was detected for major neurotransmitters involved in MSN function, including dopamine, glutamate, and GABA (Figures 2B and S3C). Dopamine and glutamate coupled with a crosstalk between PKA and CDK5 kinases are major regulators of DARPP32 phosphorylation in MSNs. Interestingly, known CTIP2 target genes concerning protein phosphorylation, serine/threonine kinase activity, Ca^2+^ transport, and cation channel activity were significantly dysregulated in our CTIP2KO datasets (Table S3) (Tang et al., 2011). Moreover, four CTIP2 target genes (*ADD3*, *CNGA3*, *DUSP16*, and *PRKCE*) appeared in the PKA gene set (Figure 2C), two of which, along with several PKA signaling genes, were validated in independent samples from all three CTIP2KO lines (Figure S3D). Dysregulation of *ADD3* and *CNGA3* was predicted to inhibit activation of cAMP-dependent PKA (Figure 3A; Table S2).

### CTIP2 Regulation of PKA-Dependent DARPP32-Thr34 and GLUR1-Ser845 Phosphorylation in MSNs

PKA is responsible for phosphorylation of DARPP32-Thr34 and GLUR1-Ser845 (Bibb et al., 1999). The predicted reduction in PKA activity from the RNA-seq analysis prompted us to investigate the phosphorylation status of DARPP32 and GLUR1. We observed an almost 50% decrease in both pDARPP32-Thr34 and pGLUR1-Ser845 levels with no change in total DARPP32 or GLUR1 protein levels in CTIP2-deficient neurons at 40 DIV (Figures 3B and 3C). When phosphorylated at Thr34, DARPP32 acts as an inhibitor of protein phosphatase 1 (PP1) by directly binding to its catalytic subunit α (PPP1CA) (Hemmings et al., 1984). Therefore, we asked whether reduced presence of pDARPP32-Thr34 in CTIP2KO MSNs had an impact on PP1 protein levels and discovered that PPP1CA content was significantly increased in CTIP2KO samples (Figures 3D and 3E).

To gain insight into potential mechanisms that led to the reduction of pDARPP32-Thr34, we explored major regulators of DARPP32 phosphorylation in MSN cultures. Protein phosphatase 3 (PP3, formerly PP2B), is known to dephosphorylate DARPP32-Thr34 in a Ca^2+^-dependent manner (Nishi et al., 1999). Western blot analysis revealed a significant increase in α (PPP3CA) and γ (PPP3CC), but not β (PPP3CB), isozymes of PP3 catalytic subunits in CTIP2-deficient MSNs (Figures 3D and 3E). Moreover, we observed significantly reduced levels of PKA catalytic subunits and elevated levels of CDK5R1, a neuron-specific activator of CDK5, in CTIP2KO cultures compared with controls (Figures 3F and 3G), although the level of CDK5 itself was not affected by CTIP2 deficiency. Our data demonstrate that the levels of MAP2, a pan neuronal protein, were similar in CTIP2KO and control cultures (Figure 3H), providing further support that the observed changes in protein kinase and phosphatase levels attribute to CTIP2 deficiency.

Finally, we demonstrate that reduction in pDARPP32-Thr34 levels is directly caused by the loss of CTIP2 in MSNs. To this extent we re-introduced CTIP2 into CTIP2KO no. 4 MSNs via viral delivery of CTIP2 transgene in postmitotic MSN neurons and determined levels of DARPP32 phosphorylation (Figures 3I and S1E). Acute restoration of CTIP2 expression in MSNs completely rescued DARPP32-Thr34 phosphorylation deficits without affecting total DARPP32 levels. Taken together these findings provide strong evidence for a role for CTIP2 in regulating PKA-dependent protein phosphorylation in MSNs.

### CTIP2-Mediated Signaling Abnormalities and Aberrant PKA-Dependent Protein Phosphorylation Are Shared by Human and Rodent HD MSNs

Because CTIP2 level is reduced in several HD models that share some of the deficits with CTIP2KO MSNs, we investigated potential overlap between CTIP2- and mHTT-mediated transcriptomic changes by comparing our MSN20 and MSN40 DEGs with four publicly available datasets of human and mouse HD models (Hodges et al., 2006, Langfelder et al., 2016, Lim et al., 2017, Ring et al., 2015). This analysis indeed revealed genes co-regulated by CTIP2 and mHTT at both time points (Table S4). We compiled a list of 1,906 and 1,976 genes from our MSN20 and MSN40 DEGs, respectively, that were concordantly dysregulated (false discovery rate < 0.01) in at least one HD gene expression dataset (Table S4). Further analysis highlighted HD signaling, oxidative phosphorylation, mitochondrial dysfunction, DNA repair (MSN20), and PKA, ERK/MAPK, and dopamine-DARPP32 feedback in cAMP signaling (MSN40) among the top 20 biological pathways affected by the co-regulated genes (Table S4). These results suggest that the signaling abnormalities caused by CTIP2 deficiency are also present in mHTT-expressing MSNs.

**Figure 4:**
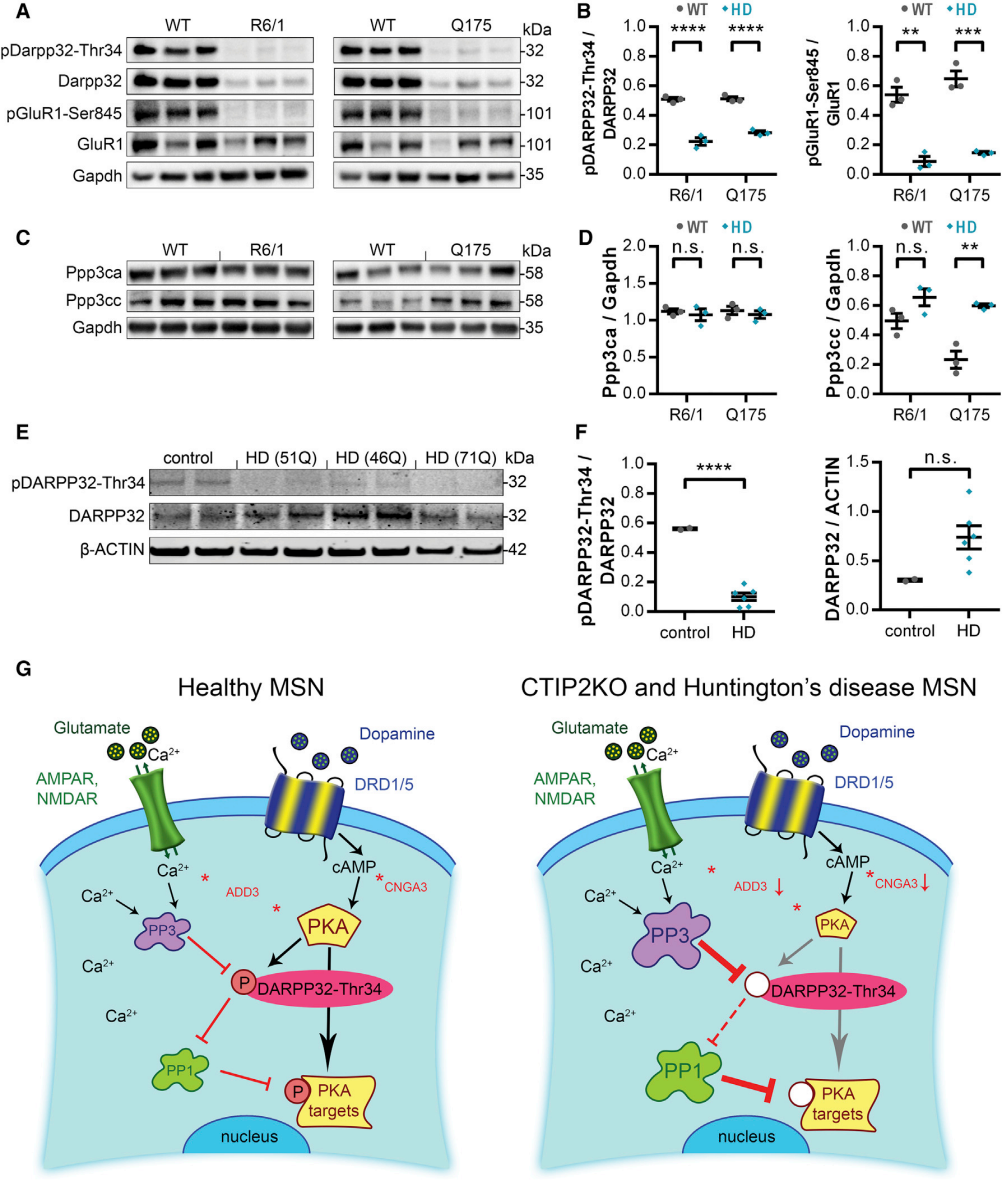
PKA-Dependent Protein Phosphorylation Deficits Are Shared by Human and Rodent mHTT-Expressing MSNs (A and B) Images (A) and quantification (B) showing a significant mHtt-dependent decrease in pDarpp32-Thr34 and pGluR1-Ser845 levels in R6/1 and Q175 HD mouse models (B) (n = 3, 3). (C and D) Images (C) and quantification (D) of two catalytic subunits of Pp3, Ppp3ca and Ppp3cc, showing a mHtt-dependent increase in Ppp3cc levels in R6/1 and Q175 mice (D) (n = 3, 3). (E and F) Images (E) and quantification (F) showing a great mHtt-dependent decrease in pDARPP32-Thr34 levels in MSNs derived from three independent HD hPSC lines (F) (n = 2, 6, 2). (B, D, and F) One-way ANOVA; ^∗∗^p < 0.01, ^∗∗∗^p < 0.001, ^∗∗∗∗^p < 0.0001; n.s., not significant. Data are presented as mean ± SEM. (G) Some of the CTIP2-regulated intracellular events are depicted in a healthy (left) versus CTIP2KO and HD (right) MSN. CTIP2 target genes in the PKA gene set are shown in red and some of their interactions with members of the PKA signaling pathway are indicated with an asterisk. Decreased PKA signaling in CTIP2KO neurons is likely mediated through lower levels of catalytic subunits of PKA available and a loss of pDARPP32-Thr34-regulated inhibition of phosphatase PP1. Most of these molecular changes are also present in mHTT-expressing cells, suggesting that CTIP2 hypofunction might contribute to selective MSN pathology in HD. See also Figure S4 and Table S4.

To strengthen this observation and provide the first experimental support, we examined DARPP32 and Glur1 phosphorylation, and Ppp3ca and Ppp3cc phosphatase levels, in animal and human cell models of HD. More than a 50% decrease in pDarpp32-Thr34 levels was observed in striatal homogenates of R6/1 and Q175 mice compared with wild-type (WT) littermates (Figures 4A and 4B). Similarly, pGluR1-Ser845 levels were greatly reduced in both HD mouse models (Figures 4A and 4B). This decrease in phosphorylation was independent of the changes in total protein levels. In line with findings in human CTIP2KO neurons, Q175 mouse striata contained significantly higher levels of Ppp3cc than WT littermates (Figures 4C and 4D). However, no significant change was observed in the levels of Ppp3ca between WT and the two HD mouse models (Figures 4C and 4D). Moreover, an 80% decrease in pDARPP32-Thr34 levels was found in MSN cultures at 40 DIV derived from three independent HD human pluripotent stem cell (hPSC) lines compared with control cells (Figures 4E and 4F). Taken together, these results suggest that CTIP2 hypofunction in striatal MSNs may contribute to neuronal cell pathologies observed in HD.

## Discussion

We have developed an efficient and versatile platform for investigating the role of transcription factor CTIP2 in human MSN development and homeostasis. CTIP2-driven DEGs identified in the current study are significantly enriched in striatal genes associated with both direct and indirect pathways in the basal ganglia, which is in line with previous findings in mice (Arlotta et al., 2008). These *Ctip2*-deficient mice exhibited normal expression of immature MSN markers (*Meis2* and *Nolz1*) in the developing LGE at embryonic day 14.5. However, the number of Darpp32^+^ neurons was significantly reduced in the newborn striatum. It remained unclear therefore whether Darpp32^+^ MSNs could initially be generated in *Ctip2*^*−/−*^ mice. Using a panel of LGE and postmitotic MSN markers, our study provides the first evidence that CTIP2 deficiency does not compromise the initial birth of MSNs from hESCs.

Moreover, this work demonstrates that CTIP2 plays a neuroprotective role against oxidative stress and is essential for maintaining striatal MSN characteristics. Notably, we reveal that CTIP2 deficiency causes an imbalance between protein kinase/phosphatase levels and activity, and subsequently reduced PKA target phosphorylation in MSNs, a cellular pathology shared by human and mouse MSNs carrying mHTT. Furthermore, our transcriptomic analysis highlights involvement of CTIP2 target genes in regulating Ca^2+^ signaling and kinase activity. *ADD3* and *CNGA3* regulate synaptic plasticity, Ca^2+^ transport, and activation of cAMP-dependent PKA, and were significantly downregulated in CTIP2-deficient MSNs (Bosia et al., 2016, Macneil et al., 1985). This provides mechanistic insight into how CTIP2 hypofunction may contribute to the reduced PKA-dependent protein phosphorylation via dysregulating Ca^2+^-mediated cAMP activity in CTIP2-deficient MSNs, and potentially in HD (Figure 4G).

PKA and CDK5 phosphorylate DARPP32 and glutamate receptors, to modulate the excitability of striatal synapses and postsynaptic signaling in MSNs, in response to dopamine and glutamate stimulation (Bibb et al., 1999). Lower PKA activity has been associated with proteasome impairments in two HD mouse models and mHTT-expressing striatal cells (Lin et al., 2013). We demonstrate reduced phosphorylation levels of PKA targets DARPP32-Thr34 and GLUR1-Ser845 in both CTIP2KO and mHTT-expressing MSNs, potentially because of inhibited activation of PKA. Furthermore, we provide evidence that DARPP32 phosphorylation deficits can be directly rescued by restoring CTIP2 levels in KO MSNs.

When phosphorylated, pDARPP32-Thr34 acts as an inhibitor of PP1, which normally dephosphorylates PKA targets (Greengard et al., 1999). Intriguingly, PP1 also regulates phosphorylation of HTT at the Thr3 site and increases its aggregation properties (Branco-Santos et al., 2017). Thus, CTIP2 hypofunction-mediated disinhibition of PP1 activity due to reduced pDARPP32-Thr34 availability would induce mHTT aggregation in HD MSNs. A higher affinity for interaction between Ctip2 and mHtt than normal Htt was demonstrated in three independent HD mouse models, and mHtt was shown to impair transcriptional function of Ctip2 (Desplats et al., 2008). Thus, abnormal kinase/phosphatase activity in CTIP2-deficient and HD MSNs, in which mHTT interacts and disrupts CTIP2 function, may underlie not only disruption of synaptic signaling in the striatum but also mHTT aggregate formation, eventually leading to MSN degeneration and cell death.

The impact of this study may go beyond the HD field because genetic mutations associated with schizophrenia were found in genes enriched in MSNs (Skene et al., 2018). Reduced levels of full-length DARPP32 and increased levels of DARPP32 isoforms lacking the crucial residue Thr34 were reported in schizophrenia patients (Kunii et al., 2014), which would have major implications for MSN regulation by dopamine. Moreover, mutations in the *CTIP2* gene, causing either CTIP2 haploinsufficiency or a truncated CTIP2 protein, have been linked to a neurodevelopmental delay with speech impairment and intellectual disability in patients (Lessel et al., 2018).

In conclusion, we have explored CTIP2-regulated molecular mechanisms in striatal neurons and demonstrated an essential role for CTIP2 in human MSN homeostasis. This study provides a robust *in vitro* framework to study neurodevelopmental phenotypes and MSN dysfunction in the context of neurological disorders, with modulation of PKA-dependent protein phosphorylation representing a potential new therapeutic target.

## Experimental Procedures

### CRISPR Design and Targeted Mutagenesis

Guide RNAs were synthesized as RNAs by *in vitro* transcription and transfected into HUES9 iCas9 hESCs as described by Gonzalez et al. (2014).

### Cell Culture and MSN Differentiation

MSNs were differentiated from the following hPSC lines: HUES9 iCas9 and genome-edited derivatives (nos. 4, 33, and 34), HD hESCs (46Q and 51Q), and HD hiPSCs (71Q; see Supplemental Experimental Procedures for HD line details). MSNs were obtained and maintained as described previously (Arber et al., 2015).

### Immunocytochemistry

Cells were incubated with primary antibodies overnight at 4°C followed by Alexa Fluor secondary antibodies for 1 h. Staining quantification was acquired manually in ImageJ (imagej.net) from >5,000 cells/sample blind to the experimental condition.

### RNA-Seq

Paired-end sequencing was performed at Oxford Genomic Center on an Illumina HiSeq 4000 (Illumina, San Diego, USA).

### Study Approval

Animal work was done under UK Home Office personal and project licenses in accordance with the requirements of the UK Animals (Scientific Procedures) Act 1986. Animals were group-housed and received water and food *ad libitum*.

### Western Blot

Equal amounts of proteins for each sample were separated on 4%–12% Bolt Bis-Tris Plus gels (Thermo Fisher Scientific) and transferred via electro-blotting to a polyvinylidene difluoride membrane (0.45 μm pore size, GE Healthcare). Membranes were incubated with primary antibodies overnight at 4°C followed by secondary antibodies for 1 h. All blot images were quantified in ImageJ blind to the experimental condition.

### Statistical Analysis

Statistical analysis was performed using one- or two-way ANOVA tests and considered statistically significant at p < 0.05. Sample sizes for each test are indicated in the figure legends as n = n_1_, n_2_, and n_3_, where n_1_ and n_2_ are the number of control and CTIP2KO (or HD) samples, respectively, from n_3_ independent experiments.

## Author Contributions

M.F. and M.L. conceived the study and designed the experiments. M.F. carried out and analyzed the CTIP2 hESC and HD animal experiments with support from Z.L. and E.D. D.C.L.F. contributed to the RNA-seq data analysis and interpretation. M.L. (Paris) and A.L.P. contributed HD hPSC data and S.P.B. supplied HD animals. M.F. and M.L. wrote the manuscript and S.P.B. edited the manuscript.

## Accession Numbers

RNA-seq data reported in this paper are available with the Sequence Read Archive under accession number SRA: SRP150394.

## Supplemental Information

Document S1. Supplemental Experimental Procedures and Figures S1–S4

Table S1. Differentially Expressed Genes in CTIP2KO MSNs at 20 and 40 DIV, Related to Figures 2 and S3

Table S2. Summary of Striatal Gene Set, KEGG Gene Set, and IPA Canonical Pathway Enrichment Analysis of CTIP2KO DEGs, Related to Figures 2, 3, and S3

Table S3. Known CTIP2 Target Genes in CTIP2KO DEGs, Related to Figures 2, 3, and S3

Table S4. Summary of HD Dataset Enrichment and Functional Annotation Analysis of the CTIP2KO-HD DEGs, Related to Figure 4

Document S2. Article plus Supplemental Information
