# Supplementary material for: CTIP2-Regulated Reduction in PKA-Dependent DARPP32 Phosphorylation in Human Medium Spiny Neurons: Implications for Huntington Disease
Source: Stem Cell Reports. 2019 Aug 22;13(3):448–57. doi: 10.1016/j.stemcr.2019.07.015 (PMC6739739; doi:10.1016/j.stemcr.2019.07.015)
Supplement: Document S1. Supplemental Experimental Procedures and Figures S1–S4 [file mmc1.pdf]

Stem Cell Reports, Volume *13*

Supplemental Information

CTIP2-Regulated Reduction in PKA-Dependent DARPP32 Phosphorylation in Human Medium Spiny Neurons: Implications for Huntington Disease

Marija Fjodorova, Morgane Louessard, Zongze Li, Daniel C. De La Fuente, Emma Dyke, Simon P. Brooks, Anselme L. Perrier, and Meng Li

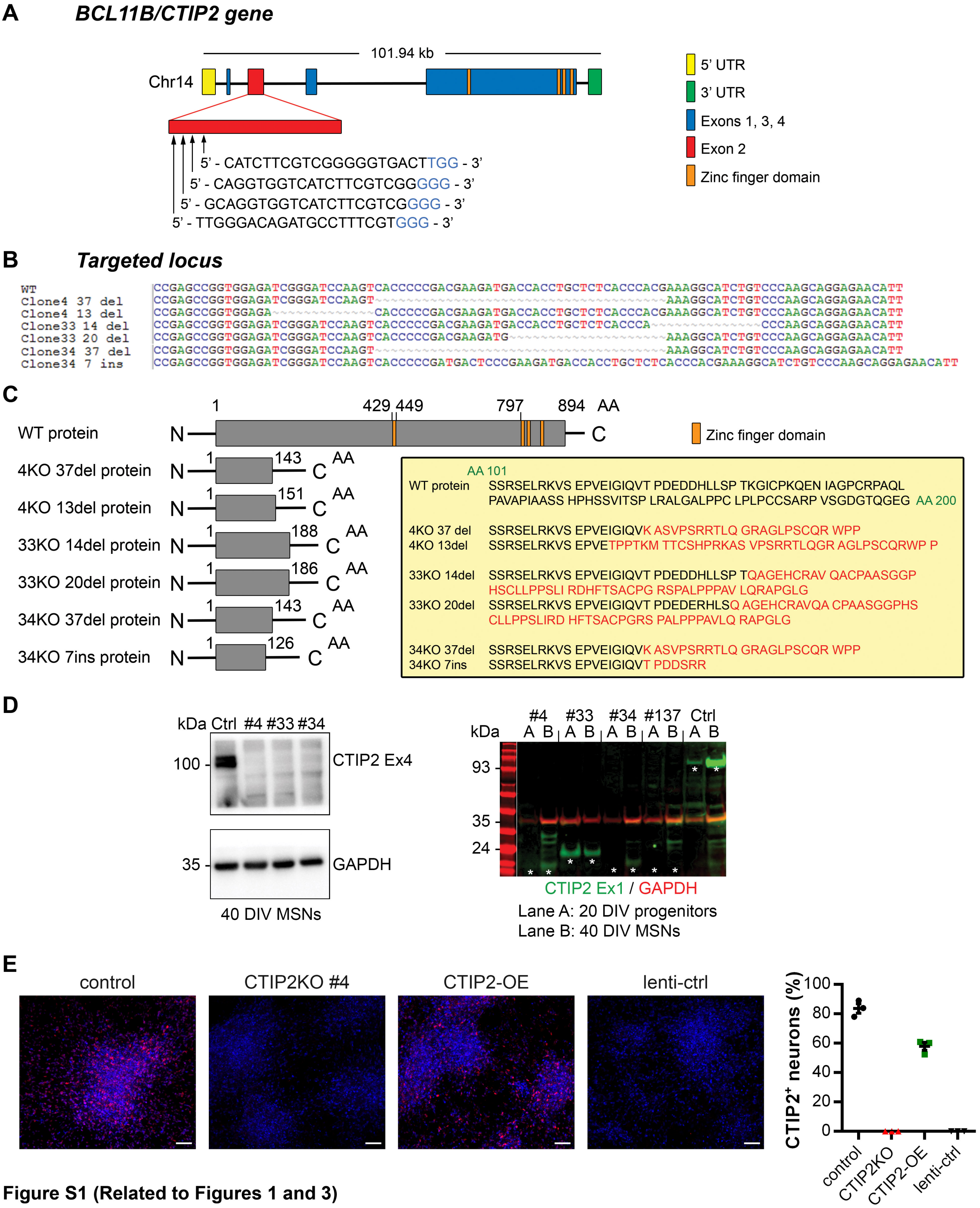

Figure S1 (Related to Figures 1 and 3)

Generation of CTIP2KO hESC lines with CRISPR/Cas9 assisted gene targeting technology. (A) Schematic illustration of the human CTIP2 gene and exon 2 targeting strategy with four gRNAs. **(B)** DNA sequences of the targeted locus in control and three independent CTIP2-/- lines (#4, #33 and #34) showing generated deletions/insertions. **(C)** Schematic illustrations and predicted amino acid sequences of truncated CTIP2 proteins resulting from frameshift mutations in CTIP2-/- lines. **(D)** Western blot analysis of CTIP2-/- lines confirms complete loss of the full-length CTIP2 protein (left) and presence of truncated CTIP2 protein isoforms (right) in CTIP2KO MSNs at 20 and 40 DIV. **(E)** Images and quantification of CTIP2^{+} cells in MSN cultures at 40DIV derived in following conditions: control, CTIP2KO #4 untreated, CTIP2KO #4 infected with either CTIP2-expressing or control lentivirus.

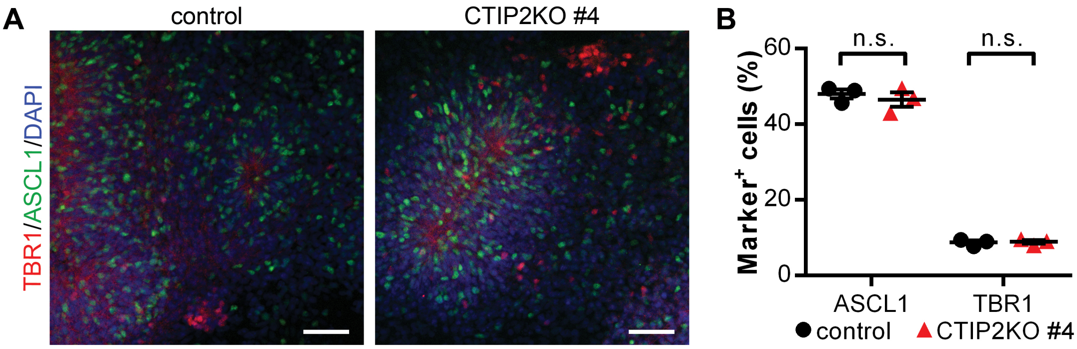

Figure S2 (Related to Figure 1)

Loss of CTIP2 does not affect regional specification of hESC-derived forebrain progenitors. Images (**A**) and quantification (**B**) of LGE-like progenitors in control and CTIP2KO #4 cultures at 20 DIV immunostained for subpallial marker ASCL1 and cortical marker TBR1. All marker+ cells were found not to differ significantly; one-way ANOVA. Mean ± s.e.m.; n.s., not significant. Scale bars: 50μm.

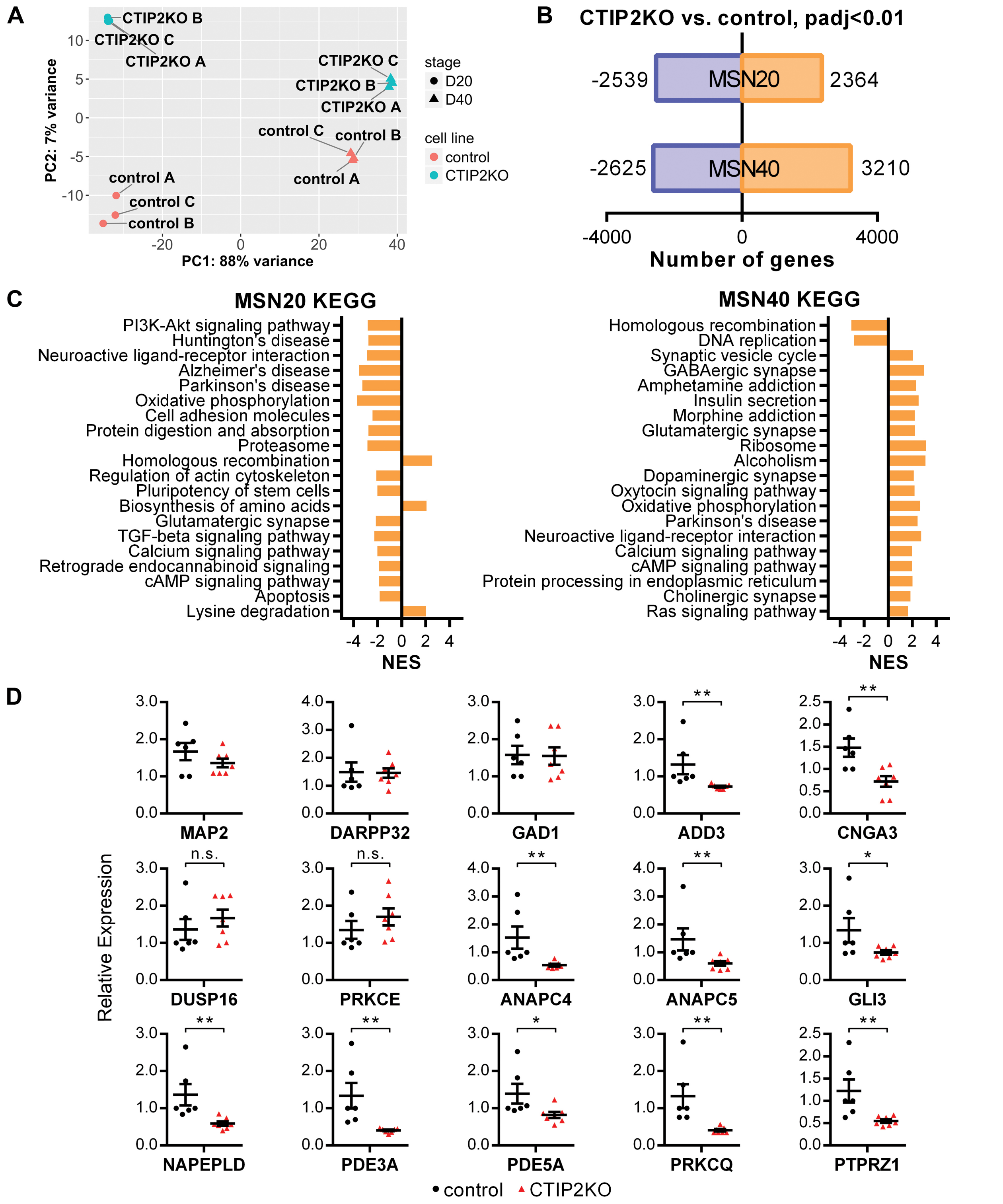

Figure S3 (Related to Figure 2)

RNA-seq analysis of D20 and D40 control and CTIP2KO MSNs with RT-PCR analysis of PKA pathway genes. **(A)** Principal component analysis of gene expression data for control and CTIP2KO #4 samples (n=3 biological replicates). **(B)** Numbers of DEGs at FDR(padj)<0.01 [upregulated genes shown in orange and downregulated genes in purple]. **(C)** KEGG pathway analysis of D20 and D40 DEGs. **(D)** RT-PCR analysis of PKA pathway genes in D40 control and CTIP2KO MSNs. One-way ANOVA or Mann-Whitney test; *,**p<0.05, 0.01; n.s., not significant [n=6,7,2].

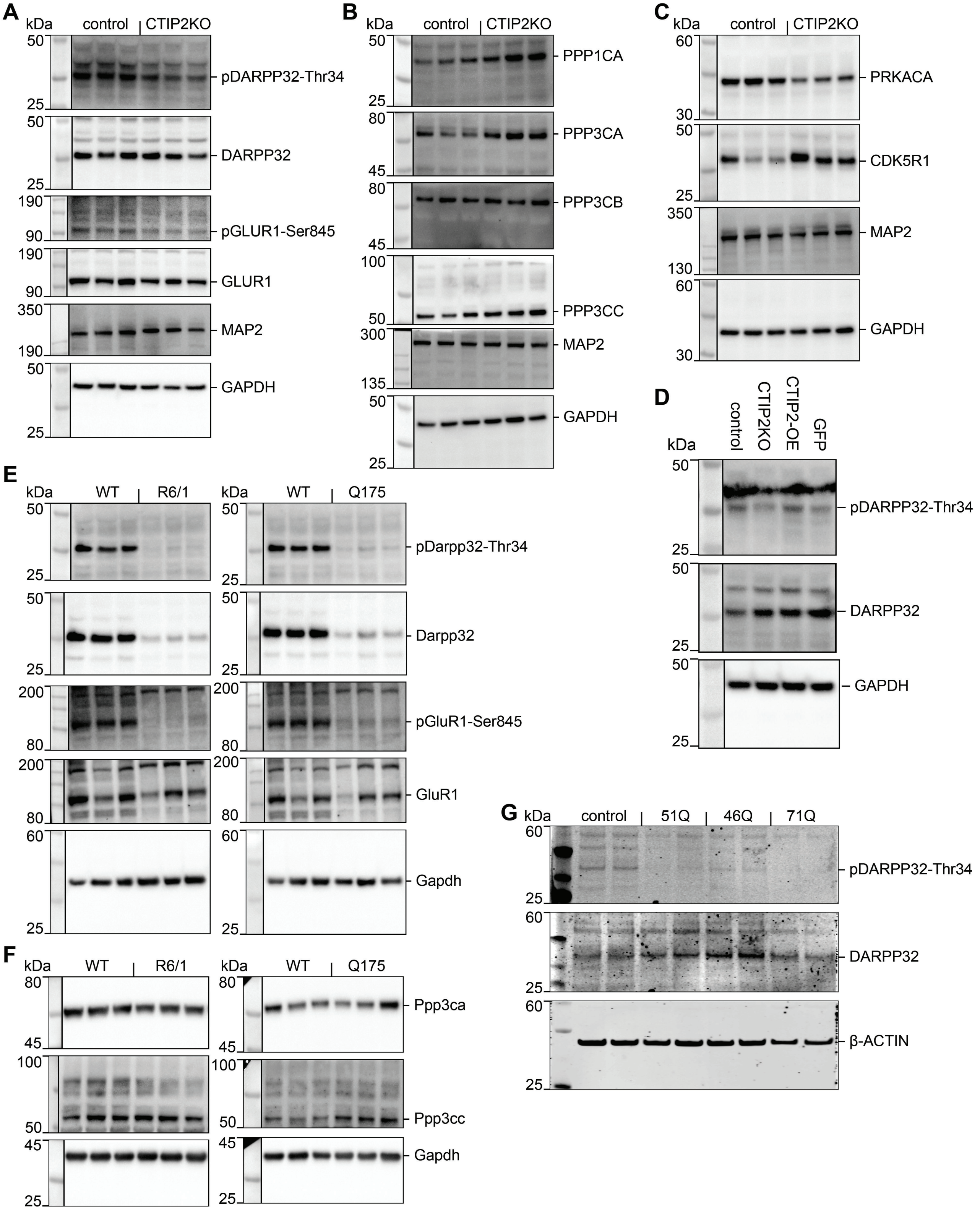

Figure S4 (Related to Figures 3 and 4)

Uncropped western blot images with markers. (A) Corresponding uncropped images of western blot shown in Figure 3B. **(B)** Corresponding uncropped images of western blot shown in Figure 3D. **(C)** Corresponding uncropped images of western blot shown in Figure 3F. **(D)** Corresponding uncropped images of western blot shown in Figure 3I. **(E)** Corresponding uncropped images of western blot shown in Figure 4A. **(F)** Corresponding uncropped images of western blot shown in Figure 4C. **(G)** Corresponding uncropped images of western blot shown in Figure 4E.

Supplemental Table Titles and Legends

Table S1 (Related to Figures 2 and S3) - Differentially expressed genes in CTIP2KO MSNs at 20 and 40 DIV. Gene lists are presented in a separate sheet for each time point (FDR<0.01). The first three columns identify the gene and the other columns contain differential expression statistics for CTIP2KO versus control group comparisons.

Table S2 (Related to Figures 2, 3 and S3) - Summary of striatal gene set, KEGG gene set and IPA canonical pathway enrichment analysis of CTIP2KO DEGs. The first sheet provides an overview and Fisher's exact test statistics for each association with striatal dataset tested. Tables #2-7 provide lists of striatum-specific MSN20 and MSN40 DEGs with differential expression statistics, each sheet corresponding to one 'time point'/'striatal dataset' combination. Tables #8-11 contain one sheet per 'time point'/analysis combination (KEGG or IPA) showing significantly dysregulated pathways (FDR<0.05), enrichment statistics and associated gene identifiers.

Table S3 (Related to Figures 2, 3 and S3) - Known CTIP2 target genes in CTIP2KO DEGs. This table contains one sheet per time point showing gene information and differential expression statistics.

Table S4 (Related to Figure 4) - Summary of HD dataset enrichment and functional annotation analysis of the CTIP2KO-HD DEGs. The first sheet provides an overview, Fisher's exact test statistics and names of dysregulated genes for each association tested. The following two sheets provide a compilation of MSN20 and MSN40 DEGs concordantly dysregulated (FDR<0.01) in at least one HD gene expression dataset. Each sheet corresponds to one time point and provides gene identifiers with differential expression statistics. Tables #4-5 contain one sheet per time point showing significantly dysregulated IPA canonical pathways (FDR<0.05), enrichment statistics and associated gene identifiers.

Supplemental Experimental Procedures

CRISPR Design and Targeted Mutagenesis

Guide RNAs (gRNAs) targeting the exon 2 of the *CTIP2* gene were selected using Zhang's lab online CRISPR Design tool (http://crispr.mit.edu/; **Figure S1A**). All gRNAs were synthesized as RNAs by *in vitro* transcription and transfected into iCas9 hESCs as described by Gonzalez *et al* (Gonzalez et al., 2014). Seven days post-transfection, individual colonies were isolated and clonally expanded. Genomic DNA was collected for each clone and the targeted region was screened by PCR (MyTaq, Bioline; forward primer: ACGCTCCGAGCTCAGGAAAG, reverse primer: GCAAGCGCAGCATCCCATAC; amplicon size: 142 bp). Genomic DNA samples showing insertions/deletions were then PCR amplified (forward primer: GTGGCCAGTGTCAAATGAAC, reverse primer: TCCTCACAGCAACCCTAATG; amplicon size: 706 bp) and cloned into a pGEM-T Easy vector (Promega) for Sanger sequencing (GATC Biotech). All studies were performed in three homozygous *CTIP2*$^{-/-}$ lines (#4, #33 and #34, referred to as CTIP2KO) and data is presented as the average between the three lines unless stated otherwise.

CTIP2 rescue

Transfer plasmid LV#1000-hPGK expressing human CTIP2 was a kind gift from Professor Malin Parmar (Lund). Production of lentivirus was performed in HEK293 cells using 2nd generation packaging system and following Lipofectamine 3000 kit guidelines (ThermoFisher Scientific). Virus was titrated in HEK cells using immunocytochemistry against CTIP2 and the resulting titer was calculated to be $9*10^9$ (U/mL). At 34 DIV, control and CTIP2KO MSNs were infected with the virus at a multiplicity of infection of 2 (MOI=number of lentiviral particles / number of target cells). At 40 DIV, cells were either fixed for immunocytochemistry or processed for protein extraction as described below.

Cell Culture and MSN Differentiation

All human pluripotent stem cell (hPSC) lines were maintained in feeder-free conditions on Matrigel-coated (Corning) plates in TeSR^{TM}-E8^{TM} medium (STEMCELL Technologies) and passaged via manual dissociation using 0.02% EDTA pH7.2 (Merck Sigma-Aldrich). Medium spiny neurons (MSNs) were differentiated from the following hPSC lines: HUES9 iCas9 (Gonzalez et al., 2014) and genome edited derivatives (#4, #33, #34), SIVF018 [HD hESCs, 46Q (Bradley et al., 2011)], SI-187 [HD hESCs, 51Q (Verlinsky et al., 2005)], ND42228 [HD hiPSCs, 71Q, RRID:CVCL_1N96 from NHCDR]. MSNs were obtained and maintained as described previously (Arber et al., 2015). In short, neural differentiation was initiated by switching TeSR^{TM}-E8^{TM} medium to DMEM-F12/Neurobasal (2:1; Thermo Fisher Scientific) supplemented with N2 (Thermo Fisher Scientific) and vitamin A-free B27 (Thermo Fisher Scientific) [referred to thereafter as N2B27] and this time point was noted as 0 days *in vitro* (0 DIV). For the first 10 DIV, cultures were supplemented with SB431542 (10 µM, Tocris), LDN-193189 (100 nM, StemGent) and dorsomorphin dihydrochloride (200 nM,Tocris). Splitting was done *en bloc* using EDTA at 9 DIV onto fibronectin-coated (Merck Millipore) plates at a ratio of 2:3. MSN fate was induced from 10 DIV by supplementing N2B27 medium with activin A (25 ng/ml, Cell Guidance Systems) until 30 DIV. Cultures were split again at 20 DIV onto poly-D-lysine hydrobromide (PDL, Merck Sigma-Aldrich) and laminin (Merck Sigma-Aldrich) co-coated plates at a density of 150k cells/cm^2. B27 with vitamin A (Thermo Fisher Scientific) was used from 20 DIV onwards. Differentiating MSNs were maintained in N2B27 supplemented with BDNF, GDNF (10ng/ml, PeproTech) from 25 DIV to aid neuronal maturation and survival.

Cytotoxicity Assay

MultiTox-Fluor Multiplex Cytotoxicity Assay (Promega) was performed following manufacturer's suggestions. Briefly, 96-well assay plates containing 40,000 MSN progenitors/well were set up at 20 DIV and cultures underwent terminal MSN differentiation. At 40 DIV, test compounds and vehicle controls were added to the cells. Treatment groups were as follows: untreated, SNAP (1000 µM) for 24 hours, amentoflavone (50 µM) for 24 hours, amentoflavone (50 µM) for 2 hours prior to addition of SNAP (1000 µM) for 24 hours (**Figure 1G**). The following controls were also included in each experiment: no cell control to determine background fluorescence, vehicle control, positive control for cytotoxicity [cells were treated with digitonin (Merck Sigma-Aldrich) at a final concentration of 50 µg/ml for 30 minutes]. Cytotoxicity assay reagents were added in an equal volume (100 µl/well) to all wells and incubated with samples for 30 minutes at 37°C. Fluorescence intensity was then measured on a microplate reader to assess viability (400Ex/505Em) and cell death (485Ex/520Em).

Immunocytochemistry

Cultured cells were rinsed in PBS and fixed in 3.7% PFA for 15 minutes. Cells were permeabilized in 0.3% Triton-X-100 solution in PBS (PBS-T) and then blocked in PBS-T containing 1% BSA and 3% donkey serum.

Cells were incubated with primary antibodies in blocking solution overnight at 4ºC. Following three PBS-T washes, Alexa-Fluor secondary antibodies (Thermo Fisher Scientific) were added at 1:1000 in blocking solution for 1 hour at ambient temperature in the dark. Cells were stained with DAPI at 1:1000 (Thermo Fisher Scientific). The following primary antibodies were used for the immunofluorescence studies: rabbit anti-GSH2 (Merck Millipore #ABN162, 1:500), goat anti-FOXP2 (Abcam #ab1307, 1:200), mouse anti-ISL1 (Developmental Studies Hybridoma Bank # 39.4D5, 1:500), goat anti-MEIS2 (Santa Cruz Biotechnology #sc-10599, 1:250), rabbit anti-FOXP2 (Abcam # ab16046, 1:500), mouse anti-FOXP1 (Abcam # ab32010, 1:800), rabbit anti-DARPP32 (Santa Cruz Biotechnology #sc-11365, 1:500), rabbit anti-TBR1 (Abcam # ab31940, 1:500), mouse anti-ASCL1 (BD Biosciences #556604, 1:500), rat anti-CTIP2 (Abcam # ab18465, 1:500). Images were taken on a Zeiss LSM710 confocal microscope from at least 5 randomly selected fields/sample and staining quantification was acquired manually in ImageJ (imagej.net) from >5,000 cells/sample blind to the experimental condition.

RNA Sequencing Data Analysis

Total RNA was extracted from TRIzol lysates using the PureLink RNA mini kit (Ambion) from three biological replicates per genotype at 20 and 40 DIV. These time points were chosen to reflect the onset of CTIP2 expression in nascent post mitotic MSNs and subsequent rapid increase in CTIP2 levels during differentiation, as well as to correspond to the time points at which several cellular pathologies have been observed. Paired-end sequencing was performed at Oxford Genomic Centre on an Illumina HiSeq 4000 (Illumina, San Diego, USA) obtaining a library size of ~80 million reads per sample. FASTQ files were trimmed and mapped to the Ensembl human genome GRCh38.84 (hg38) using STAR (v2.5.1b). Quality of the samples was assessed using FastQC (v0.11.2) prior to and after trimming. Gene counts were obtained from the number of uniquely aligned unambiguous reads by Subread:featureCount (v1.4.6-p2). Library size normalization and differentially expressed genes (DEGs) were determined using the R/Bioconductor package DESeq2 (v1.14.1) (Love et al., 2014). Benjamini-Hochberg (BH) test for multiple correction was used to control the false discovery rate (FDR). Subsequent study of KEGG gene set and pathway enrichment analysis was performed on protein coding DEGs with an EntrezID and FDR<0.01 using the Ingenuity Pathway Analysis (Qiagen) and R/Bioconductor package clusterProfiler (v3.12.14) (Yu et al., 2012). The associations between present CTIP2KO gene sets and published striatal and HD gene sets were determined by Fisher's exact test, performed in RStudio (www.r-project.org).

PCR Analysis

Total mRNA was extracted from TRIzol lysates using the PureLink RNA mini kit (Ambion) from three biological replicates per line at 40 DIV. RT-PCR was completed using 2 ug mRNA and the EvoScript kit (Roche). MesaGreen kit was used to perform qPCR using 200 pg mRNA/reaction to validate CTIP2KO DEGs in the PKA gene set. The following primers were used:

Gene Name	Forward Primer	Reverse Primer
MAP2	CAACGGAGAGCTGACCTCA	CTACAGCCTCAGCAGTGACTA
DARPP32	GGTATTTTTATCCGTGCGCGAAC	CTTCCTCCTCTGGTGAGGAGTG
GAD1	CGAGGACTCTGGACAGTAGAGG	GATCTTGAGCCCCAGTTTTCTG
ADD3	CAGCCAAGGCGTGATTACCA	TCTTCCCGAAAGGCAGGACT
CNGA3	GGGACCGGACTCTTTTCCTG	CACCACGATCGCATCCTTCT
DUSP16	ACGATCAAAGCTCCCAAGATGT	GCCAGGGAAACAACGAGAGA
PRKCE	CAACGGCCACAAGTTCATGG	AGGTGCAGACTTGACACTGG
ANAPC4	ATTTTCCTGGTCTGGTCGCC	CCAGACACGTCACCTCCTTT
ANAPC5	GGTGCTGCTGAACGAGATGAG	GAATTTGCCAGCTGTGGACAAG
GLI3	GCCTCCAGCACCACTTCTAAT	TCAATGAGGCCCTCTCGTCA
NAPEPLD	TCGGAGCTTATGAACCGAGG	AGGCAAAAGTTCCCCAGTGA
PDE3A	ACAGGTCTACCCACCTTGGA	AGGATCTGCTTTTGGTGAGGG
PDE5A	TGGTGAGCCCTTGAACATCA	CTGGGCTACACCAACAACCT
PRKCQ	AAAGGTCCACCACGTCAAGT	GTAGCCCTGTTTGTTCAGGC
PTPRZ1	TTGTTGAAGAGATTGGCTGGTC	TTGATAGGAGATTGTTTTGGGCT

Study Approval

All animal work was done under UK Home Office personal and project licenses in accordance with the requirements of the UK Animals (Scientific Procedures) Act 1986 and European Directive 2010/63/EU and with the approval of the local Cardiff University Ethics Review Committee. Animals were group-housed and received water and food *ad libitum*.

Western Blot Analysis

At 40 DIV, cells were collected in PBS and lysed on ice in RIPA buffer (Abcam) supplemented with protease and phosphatase inhibitors (Merck Sigma-Aldrich). Striatal region was dissected out from adult heterozygous HD mice and respective WT littermates (R6/1: 5 months, Q175: 24 months). Tissues were triturated by pipetting and lysed as described above. Protein concentrations of whole cell lysates were measured using the DC protein assay kit (BioRad) and then combined with 1X Bolt LDS Sample Buffer (Thermo Fisher Scientific) and 1X Bolt Sample Reducing Agent (Thermo Fisher Scientific). Equal amounts of proteins for each sample were separated on 4-12% Bolt Bis-Tris Plus gels (Thermo Fisher Scientific) and transferred via electro-blotting to a PVDF membrane (0.45 μm pore size, GE Healthcare). Membranes were blocked with 5% BSA in TBS containing 0.1% Tween-20 (TBS-T) for 2 hours at ambient temperature and then incubated with primary antibodies overnight at 4°C. The following primary antibodies were used for the western blot studies: rabbit anti-pDARPP32-Thr34 (Cell Signaling Technology #12438-D27A4, 1:1000), rabbit anti-DARPP32 (Santa Cruz Biotechnology #sc-11365, 1:500), rabbit anti-pGLUR1-Ser845 (Cell Signaling Technology #8084-D10G5, 1:1000), rabbit anti-GLUR1 (Cell Signaling Technology #13185-D4N9V, 1:1000), mouse anti-PP1α (PPP1CA, Santa Cruz Biotechnology #sc-271762, 1:1000), mouse anti-PP2B-Aα (PPP3CA, Santa Cruz Biotechnology #sc-17808, 1:1000), mouse anti-PP2B-Aβ (PPP3CB, Santa Cruz Biotechnology #sc-365612, 1:1000), mouse anti-PP2B-Aγ (PPP3CC, Santa Cruz Biotechnology #sc-293361, 1:1000), rabbit anti-PKA catalytic subunit (PKAcat, Abcam #ab76238, 1:5000), rabbit anti-p35/25 (CDK5R1, Cell Signaling Technology #2680-C64B10, 1:1000), rabbit anti-MAP2 (Merck Sigma-Aldrich #AB5622, 1:1000), rabbit anti-CTIP2 (Abcam #ab70453, 1:2000), rat anti-CTIP2 (Abcam # ab18465, 1:1000), mouse anti-GAPDH (Abcam #8245, 1:10000), mouse anti-β-ACTIN (Merck Sigma-Aldrich #A3854, 1:50000). Following three TBS-T washes, membranes were incubated with a horseradish peroxidase-conjugated anti-mouse or anti-rabbit antibody (Abcam) for 1 hour at ambient temperature. For CTIP2/GAPDH blotting (**Figure S1D**), membranes were incubated with anti-rat IRDye 800CW and anti-mouse IRDye 680RD secondary antibodies (LI-COR). Blots were developed with ECL system (Thermo Fisher Scientific) and imaged on an Odyssey CLx imaging system (LI-COR) or on a Gel Doc XR system (Bio-Rad). All images were quantified in ImageJ blind to the experimental condition.

Statistical Analysis

RNA sequencing data was analyzed separately as described above. SPSS Statistics 20 software (IBM) was used for all other statistical analyses. All quantified data were plotted in Prism 6 (GraphPad Software) and are reported as mean ± SEM with sample sizes for each test indicated in the figure legends. Horizontal line on dot plots depicts mean ± SEM for each genotype, with the means for individual clones indicated by red-shaded circles beside CTIP2KO data. Normal distribution of data was assessed the Shapiro-Wilk test. Statistical analysis was performed using one-/two-way ANOVA tests followed by Bonferroni's correction for multiple comparisons if applicable or non-parametric Mann-Whitney test for not normally distributed data. Results were considered statistically significant at $p<0.05$. No statistical methods were used to predetermine sample sizes, but our sample sizes are similar to those reported in previous publications (Arber et al., 2015; Cavey et al., 2016; Victor et al., 2018). Randomization was used to assign experimental conditions and collect data, and data collection was always done in parallel with controls. Data analyses were not performed blind to the conditions of the experiments except where stated otherwise.

Data and Software Availability

RNA-seq data reported in this paper are available with the SRA accession number **SRP150394** (https://www.ncbi.nlm.nih.gov/sra/SRP150394). No custom algorithms were used to analyze data in this study. The authors declare that all data supporting the findings of this study are available within this article, its Supplemental Information files, or are available from the corresponding authors upon reasonable request.

Supplemental References

Arber, C., Precious, S.V., Cambray, S., Risner-Janiczek, J.R., Kelly, C., Noakes, Z., Fjodorova, M., Heuer, A., Ungless, M.A., Rodriguez, T.A.*, et al.* (2015). Activin A directs striatal projection neuron differentiation of human pluripotent stem cells. Development *142*, 1375-1386.

Cavey, M., Collins, B., Bertet, C., and Blau, J. (2016). Circadian rhythms in neuronal activity propagate through output circuits. Nat. Neurosci. *19*, 587-595.

Gonzalez, F., Zhu, Z., Shi, Z.-D., Lelli, K., Verma, N., Li, Q.V., and Huangfu, D. (2014). An iCRISPR Platform for Rapid, Multiplexable, and Inducible Genome Editing in Human Pluripotent Stem Cells. Cell Stem Cell *15*, 215-226.

Love, M.I., Huber, W., and Anders, S. (2014). Moderated estimation of fold change and dispersion for RNA-seq data with DESeq2. Genome Biol. *15*, 550.

Victor, M.B., Richner, M., Olsen, H.E., Lee, S.W., Monteys, A.M., Ma, C., Huh, C.J., Zhang, B., Davidson, B.L., Yang, X.W., *et al.* (2018). Striatal neurons directly converted from Huntington's disease patient fibroblasts recapitulate age-associated disease phenotypes. Nat. Neurosci. *21*, 341-352.
Yu, G., Wang, L.-G., Han, Y., and He, Q.-Y. (2012). clusterProfiler: an R Package for Comparing Biological Themes Among Gene Clusters. OMICS *16*, 284-287.
