# Supplementary material for: CTIP2-Regulated Reduction in PKA-Dependent DARPP32 Phosphorylation in Human Medium Spiny Neurons: Implications for Huntington Disease
Source: Stem Cell Reports. 2019 Aug 22;13(3):448–57. doi: 10.1016/j.stemcr.2019.07.015 (PMC6739739; doi:10.1016/j.stemcr.2019.07.015)
Supplement: Document S2. Article plus Supplemental Information [file mmc6.pdf]

Stem Cell Reports
Report
ISSCR
OPEN ACCESS

CTIP2-Regulated Reduction in PKA-Dependent DARPP32 Phosphorylation in Human Medium Spiny Neurons: Implications for Huntington Disease

Marija Fjodorova,[1,*] Morgane Louessard,[3] Zongze Li,[1] Daniel C. De La Fuente,[1] Emma Dyke,[1] Simon P. Brooks,[2] Anselme L. Perrier,[3] and Meng Li[1,2,*]
[1]Neuroscience and Mental Health Research Institute, School of Medicine, Cardiff University, Cardiff CF24 4HQ, UK
[2]Division of Neuroscience, School of Bioscience, Cardiff University, Cardiff CF10 3AX, UK
[3]Institut National de la Santé et de la Recherche Médicale (INSERM) UMR861, I-Stem, AFM, 91100 Corbeil-Essonnes, France
*Correspondence: fjodorovam@cardiff.ac.uk (M.F.), lim26@cardiff.ac.uk (M.L.)
https://doi.org/10.1016/j.stemcr.2019.07.015

SUMMARY

The mechanisms underlying the selective degeneration of medium spiny neurons (MSNs) in Huntington disease (HD) remain largely unknown. CTIP2, a transcription factor expressed by all MSNs, is implicated in HD pathogenesis because of its interactions with mutant huntingtin. Here, we report a key role for CTIP2 in protein phosphorylation via governing protein kinase A (PKA) signaling in human striatal neurons. Transcriptomic analysis of CTIP2-deficient MSNs implicates CTIP2 target genes at the heart of cAMP-Ca^{2+} signal integration in the PKA pathway. These findings are further supported by experimental evidence of a substantial reduction in phosphorylation of DARPP32 and GLUR1, two PKA targets in CTIP2-deficient MSNs. Moreover, we show that CTIP2-dependent dysregulation of protein phosphorylation is shared by HD hPSC-derived MSNs and striatal tissues of two HD mouse models. This study therefore establishes an essential role for CTIP2 in human MSN homeostasis and provides mechanistic and potential therapeutic insight into striatal neurodegeneration.

INTRODUCTION

Inhibitory γ-amino butyric acid (GABA)-releasing medium spiny neurons (MSNs) are the principal projection neurons of the basal ganglia, receiving inputs from both glutamatergic cortical neurons and midbrain dopaminergic neurons. DARPP32 is a class-defining protein marker for striatal MSNs and a central mediator of dopaminergic and other first-messenger signaling in these cells. Specifically, phosphorylation of DARPP32 at threonine 34 (pDARPP32-Thr34) by protein kinase A (PKA), following dopamine D1 receptor activation, is critically involved in regulating electrophysiological, transcriptional and behavioral responses of MSNs to physiological and pharmacological stimuli, including antidepressants, neuroleptics, and drugs of abuse (Yger and Girault, 2011). Thus, these findings point to an important role for PKA-regulated phosphorylation of DARPP32 in MSN health, with major implications for neurological disorders.

CTIP2 (also known as BCL11B) is a transcription factor expressed by all MSNs and is required for MSN development and transcriptional regulation of striatal genes (Arlotta et al., 2008; Onorati et al., 2014). CTIP2 deficiency results in structural striatal defects, impaired spatial learning, and working memory deficits in mice (Arlotta et al., 2008; Simon et al., 2016). Furthermore, CTIP2 protein levels are reduced in both human and rodent mutant huntingtin (mHTT)-expressing cells before the onset of MSN degeneration (Langfelder et al., 2016; Ring et al., 2015), pointing to a role for CTIP2 in Huntington disease (HD) pathogenesis. These findings have led us to hypothesize that CTIP2 may play an important role in conferring regional specificity of HD neurodegeneration that cannot be explained by the ubiquitous expression of the mHTT. However, whether CTIP2 deficiency directly leads to increased MSN vulnerability and dysfunction remains to be demonstrated.

Using CRISPR/Cas9 genome-edited human embryonic stem cells (hESCs) as a model, we uncover a role for CTIP2 in regulating protein kinase and phosphatase levels and activity and subsequently phosphorylation of DARPP32-Thr34 in MSNs. We show for the first time that deficits in PKA-dependent protein phosphorylation occur in human and mouse HD MSNs, potentially owing to CTIP2- and mHTT-co-regulated molecular signaling abnormalities as suggested by transcriptomic analysis. This study provides evidence of a central role for CTIP2 in human MSN homeostasis and supports the hypothesis that CTIP2 may mediate regional specificity of HD pathogenesis.

RESULTS

CTIP2 Deficiency Does Not Compromise the Generation of MSNs from hESCs

Homozygous CTIP2 knockout (KO) hESC lines were generated using the CRISPR/Cas9-assisted genome editing technology with guide RNAs targeting exon 2 of the *CTIP2* gene (Figures S1A–S1D). To investigate whether CTIP2 deficiency affects the generation of MSNs from hESCs, we

This is an open access article under the CC BY license (http://creativecommons.org/licenses/by/4.0/).

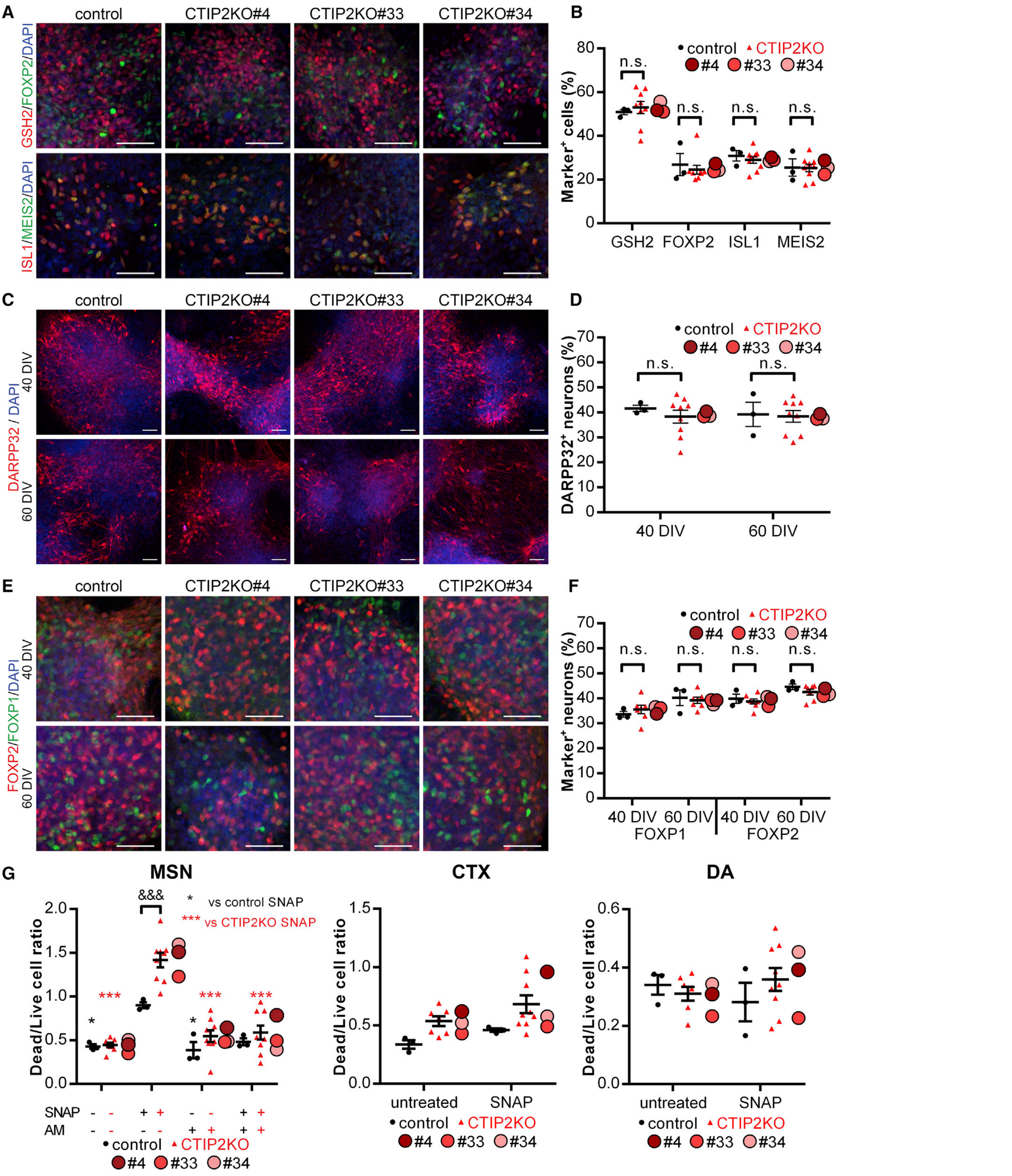

(legend on next page)

performed striatal neural differentiation on CTIP2KO and control lines using an established protocol (Arber et al., 2015). At 20 days *in vitro* (DIV), both control and CTIP2KO hESCs gave rise to high yields of cells expressing lateral ganglionic eminence (LGE) markers GSX2, FOXP2, ISL1, and MEIS2, subpallial marker ASCL1, with very few TBR1$^+$ cortical cells (Figures 1A, 1B, and S2). These LGE-like progenitors differentiated into 35%–40% DARPP32$^+$, FOXP1$^+$, and FOXP2$^+$ MSNs by 40 DIV and maintained MSN identity for the subsequent 20-day period (Figures 1C–1F). Taken together, these results suggest that the loss of CTIP2 does not affect the yield of nascent and mature MSNs derived from hESCs.

CTIP2-Deficient MSNs Display Increased Vulnerability to Oxidative Stress

We then asked whether loss of CTIP2 would compromise MSN health. To this extent we investigated oxidative stress-dependent cell death using the nitric oxide donor *S*-nitroso-*N*-acetylpenicillamine (SNAP), which was previously shown to generate reactive oxygen species and induce oxidative stress (Wei et al., 2000). After a 24-h exposure of cells to SNAP we observed a 2-fold greater increase in apoptosis in CTIP2KO MSNs compared with control neurons (Figure 1G). This phenotype was rescued by a 2-h pre-treatment with the neuroprotective and anti-apoptotic agent amentoflavone (Figure 1G, MSN), a drug previously shown to reduce neuronal damage via decreasing nitric oxide production in response to apoptotic stimuli (Zhang et al., 2015). We next checked if CTIP2-dependent cell death deficits were MSN specific by analyzing differentiated CTIP2-expressing cortical neurons and dopamine neurons that do not express CTIP2. Interestingly, cortical neurons were less vulnerable to oxidative stress than MSNs, with CTIP2KO neurons showing only a trend toward a more severe phenotype than control (Figure 1G, CTX). Oxidative stress-dependent cell death levels were not affected by CTIP2KO in dopaminergic neurons (Figure 1G, DA). This data suggests that CTIP2 plays a neuroprotective role against oxidative stress that is restricted to MSNs.

Genome-wide Transcriptome Analysis Highlights a Role for CTIP2 in PKA-Regulated Protein Phosphorylation

To investigate the molecular mechanisms and pathways leading to pathological changes in CTIP2-deficient MSNs, we performed whole-transcriptome RNA sequencing (RNA-seq) analysis of CTIP2KO no. 4 and control MSN cultures at 20 and 40 DIV (MSN20 and MSN40, respectively). Global principal component analysis showed clear sample segregation based on genotype and developmental stage (Figure S3A). Analysis of protein-coding genes identified 4,903 and 5,835 differentially expressed genes (DEGs) for MSN20 and MSN40, respectively (Figure S3B; Table S1). Moreover, MSN20 and MSN40 DEGs were enriched for human striatum-specific genes and significantly associated with the Ctip2 loss-of-function study in mouse striatum (Arlotta et al., 2008; Onorati et al., 2014) (Table S2). Among these striatal genes are *EBF1*, *LMO3*, *CNR1*, *DRD1*, *DRD2*, *SST*, *REC8*, *GRM1*, and *PDE5A*, which were all downregulated in CTIP2KO cells.

We identified mitochondrial dysfunction, oxidative phosphorylation, calcium signaling, and HD signaling among the significantly dysregulated pathways at both time points (Figures 2A, 2B, and S3C; Table S2). Intriguingly, genes concerning dopamine-DARPP32 feedback in cAMP signaling, PKA and CDK5 signaling were found to be significantly altered in mature MSNs (Figure 2B). Also, significant dysregulation of synaptic signaling was detected for major neurotransmitters involved in MSN function, including dopamine, glutamate, and GABA (Figures 2B and S3C). Dopamine and glutamate coupled with a crosstalk between PKA and CDK5 kinases are major regulators of DARPP32 phosphorylation in MSNs. Interestingly, known CTIP2 target genes concerning protein phosphorylation, serine/threonine kinase activity, Ca^{2+} transport, and cation channel activity were significantly dysregulated in our CTIP2KO datasets (Table S3) (Tang et al., 2011). Moreover, four CTIP2 target genes (*ADD3*, *CNGA3*, *DUSP16*, and *PRKCE*) appeared in the PKA gene set (Figure 2C), two of which, along with several PKA signaling genes,

Figure 1. CTIP2KO MSNs Acquire Normal Striatal Cell Identity but Present with Increased Vulnerability to Oxidative Stress

(A and B) LGE-like progenitors in control and CTIP2KO cultures at 20 DIV labeled and quantified for GSH2, FOXP2, ISL1, and MEIS2 (n = 3, 9, 3).

(C and D) MSNs in control and CTIP2KO cultures at 40 and 60 DIV labeled and quantified for DARPP32 (n = 3, 9, 3).

(E and F) MSNs in control and CTIP2KO cultures at 40 and 60 DIV labeled and quantified for FOXP1 and FOXP2 (n = 3, 7, $\geq$2).

(G) Pre-treatment of MSNs with 50 μM amentoflavone (AF) for 2 h protects them from SNAP-induced cell death at 40 DIV. Similar vulnerability to oxidative stress is observed between control and CTIP2-deficient groups in both cortical neurons (CTX) and dopaminergic (DA) neurons (n = 3, 9, 3).

(A, C, and E) Scale bars, 50 μm. (B, D, and F) One-way ANOVA; n.s., not significant. (G) Two-way ANOVA; MSN: $^*p < 0.05$, $^{***}p < 0.001$; $^{\&\&\&}p < 0.001$. Data are presented as mean ± SEM for each genotype, with the means for individual clones indicated by red-shaded circles beside CTIP2KO data.

See also Figures S1 and S2.

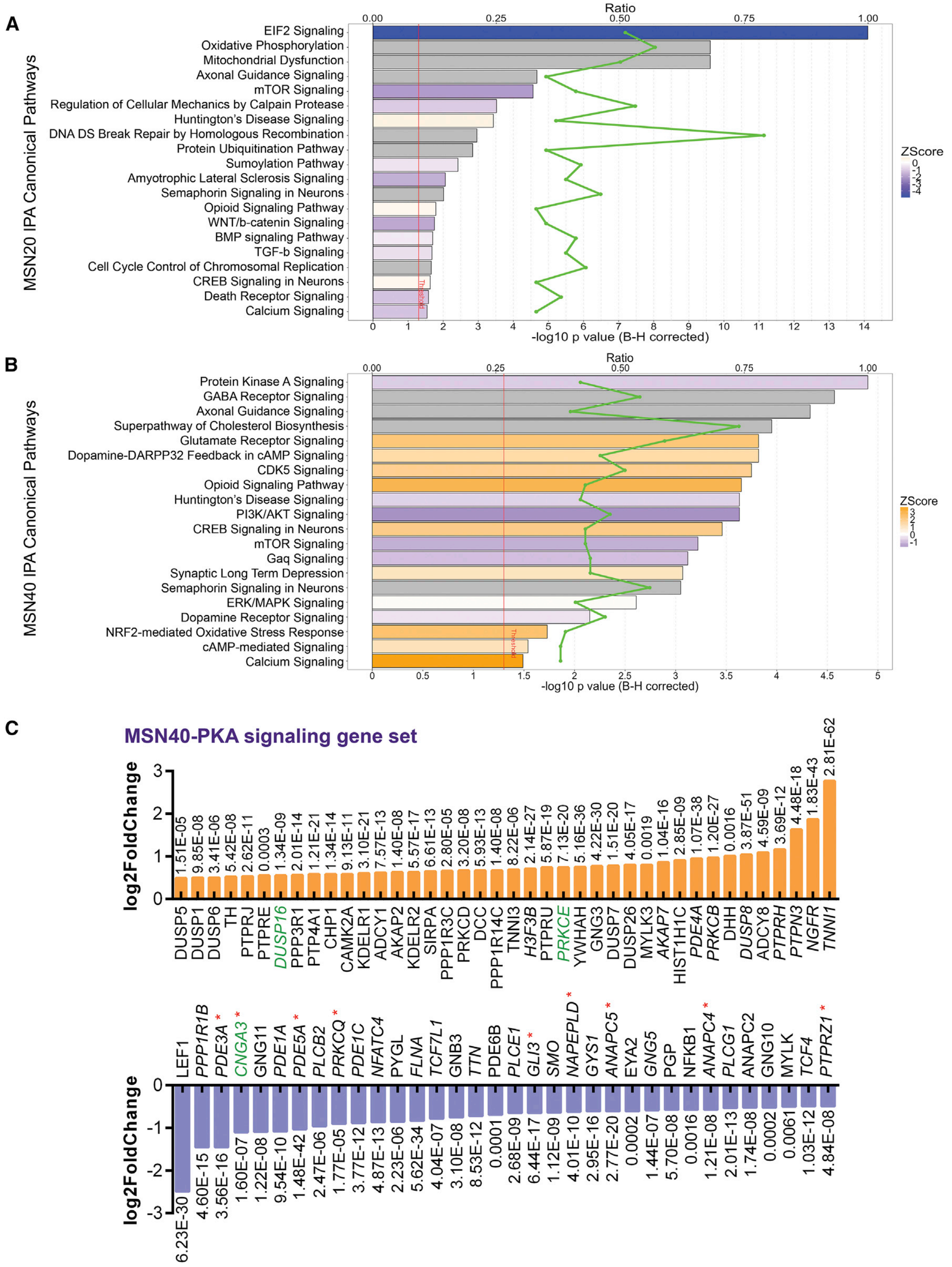

(legend on next page)

were validated in independent samples from all three CTIP2KO lines (Figure S3D). Dysregulation of *ADD3* and *CNGA3* was predicted to inhibit activation of cAMP-dependent PKA (Figure 3A; Table S2).

CTIP2 Regulation of PKA-Dependent DARPP32-Thr34 and GLUR1-Ser845 Phosphorylation in MSNs

PKA is responsible for phosphorylation of DARPP32-Thr34 and GLUR1-Ser845 (Bibb et al., 1999). The predicted reduction in PKA activity from the RNA-seq analysis prompted us to investigate the phosphorylation status of DARPP32 and GLUR1. We observed an almost 50% decrease in both pDARPP32-Thr34 and pGLUR1-Ser845 levels with no change in total DARPP32 or GLUR1 protein levels in CTIP2-deficient neurons at 40 DIV (Figures 3B and 3C). When phosphorylated at Thr34, DARPP32 acts as an inhibitor of protein phosphatase 1 (PP1) by directly binding to its catalytic subunit α (PPP1CA) (Hemmings et al., 1984). Therefore, we asked whether reduced presence of pDARPP32-Thr34 in CTIP2KO MSNs had an impact on PP1 protein levels and discovered that PPP1CA content was significantly increased in CTIP2KO samples (Figures 3D and 3E).

To gain insight into potential mechanisms that led to the reduction of pDARPP32-Thr34, we explored major regulators of DARPP32 phosphorylation in MSN cultures. Protein phosphatase 3 (PP3, formerly PP2B), is known to dephosphorylate DARPP32-Thr34 in a Ca^{2+}-dependent manner (Nishi et al., 1999). Western blot analysis revealed a significant increase in α (PPP3CA) and γ (PPP3CC), but not β (PPP3CB), isozymes of PP3 catalytic subunits in CTIP2-deficient MSNs (Figures 3D and 3E). Moreover, we observed significantly reduced levels of PKA catalytic subunits and elevated levels of CDK5R1, a neuron-specific activator of CDK5, in CTIP2KO cultures compared with controls (Figures 3F and 3G), although the level of CDK5 itself was not affected by CTIP2 deficiency. Our data demonstrate that the levels of MAP2, a pan neuronal protein, were similar in CTIP2KO and control cultures (Figure 3H), providing further support that the observed changes in protein kinase and phosphatase levels attribute to CTIP2 deficiency.

Finally, we demonstrate that reduction in pDARPP32-Thr34 levels is directly caused by the loss of CTIP2 in MSNs. To this extent we re-introduced CTIP2 into CTIP2KO no. 4 MSNs via viral delivery of CTIP2 transgene in postmitotic MSN neurons and determined levels of DARPP32 phosphorylation (Figures 3I and S1E). Acute restoration of CTIP2 expression in MSNs completely rescued DARPP32-Thr34 phosphorylation deficits without affecting total DARPP32 levels. Taken together these findings provide strong evidence for a role for CTIP2 in regulating PKA-dependent protein phosphorylation in MSNs.

CTIP2-Mediated Signaling Abnormalities and Aberrant PKA-Dependent Protein Phosphorylation Are Shared by Human and Rodent HD MSNs

Because CTIP2 level is reduced in several HD models that share some of the deficits with CTIP2KO MSNs, we investigated potential overlap between CTIP2- and mHTT-mediated transcriptomic changes by comparing our MSN20 and MSN40 DEGs with four publicly available datasets of human and mouse HD models (Hodges et al., 2006; Langfelder et al., 2016; Lim et al., 2017; Ring et al., 2015). This analysis indeed revealed genes co-regulated by CTIP2 and mHTT at both time points (Table S4). We compiled a list of 1,906 and 1,976 genes from our MSN20 and MSN40 DEGs, respectively, that were concordantly dysregulated (false discovery rate < 0.01) in at least one HD gene expression dataset (Table S4). Further analysis highlighted HD signaling, oxidative phosphorylation, mitochondrial dysfunction, DNA repair (MSN20), and PKA, ERK/MAPK, and dopamine-DARPP32 feedback in cAMP signaling (MSN40) among the top 20 biological pathways affected by the co-regulated genes (Table S4). These results suggest that the signaling abnormalities caused by CTIP2 deficiency are also present in mHTT-expressing MSNs.

To strengthen this observation and provide the first experimental support, we examined DARPP32 and Glur1 phosphorylation, and Ppp3ca and Ppp3cc phosphatase levels, in animal and human cell models of HD. More than a 50% decrease in pDarpp32-Thr34 levels was observed in striatal homogenates of R6/1 and Q175 mice compared with wild-type (WT) littermates (Figures 4A and 4B). Similarly, pGluR1-Ser845 levels were greatly reduced in both HD mouse models (Figures 4A and 4B). This decrease in phosphorylation was independent of the changes in total protein levels. In line with findings

Figure 2. RNA-Seq Analysis Highlights a Role for CTIP2 in PKA-Regulated Protein Phosphorylation in hESC-Derived MSNs

(A and B) Ingenuity pathway analysis (IPA) of MSN20 (A) and MSN40 (B) CTIP2KO DEGs shows significant enrichment of genes regulating mitochondrial function at 20 DIV, PKA and CDK5 signaling as well as dopamine-DARPP32 feedback in cAMP signaling at 40 DIV (full gene set lists for both time points are presented in Table S2).

(C) Differential expression statistics for MSN40 DEGs within the PKA gene set at fold change threshold of |1.4|, with direct CTIP2 targets highlighted in green. Genes analyzed by RT-PCR in independent control and CTIP2KO MSN samples are indicated in italic, with red asterisks marking validated differential expression.

See also Figure S3 and Tables S2 and S3.

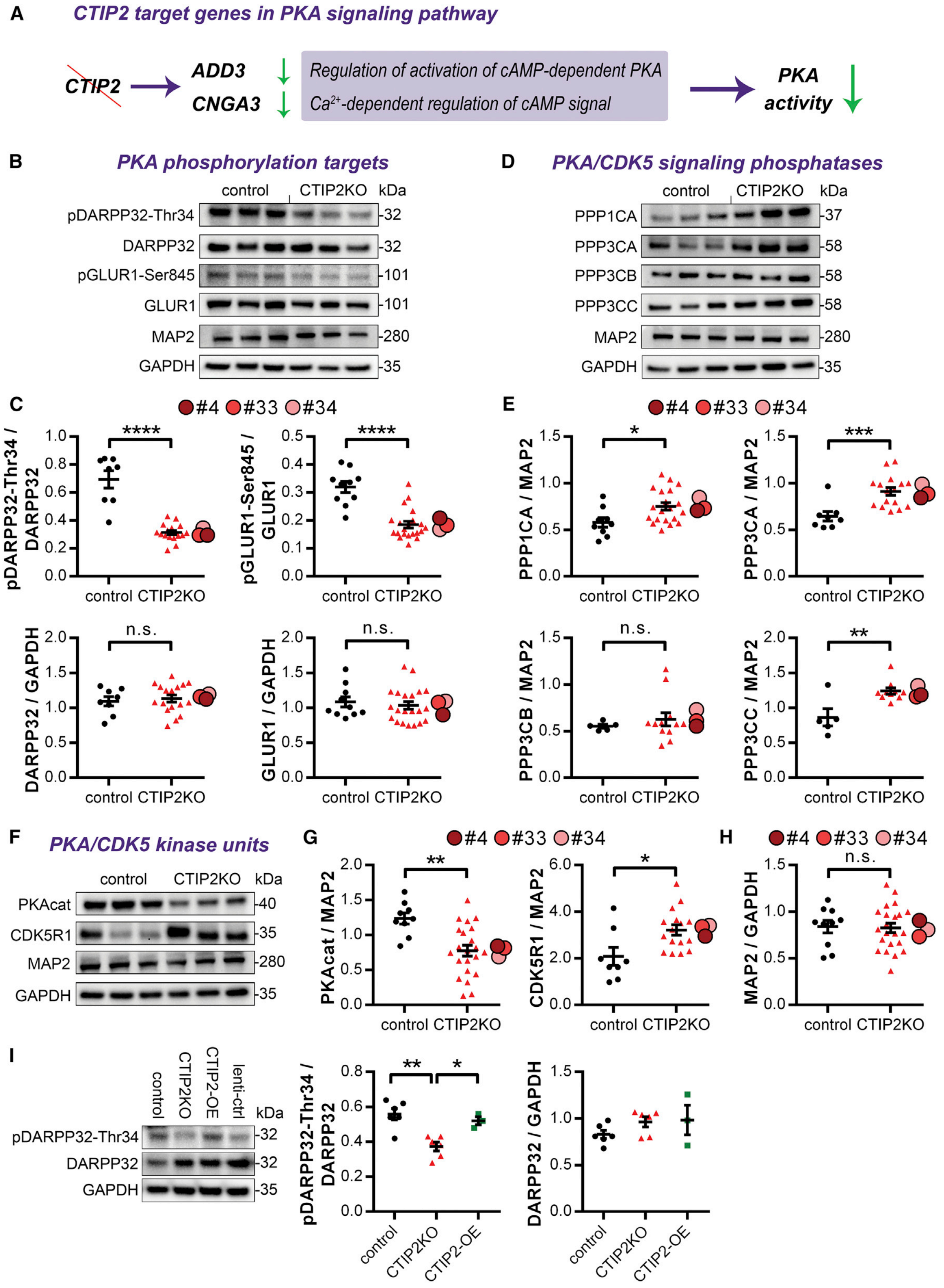

(legend on next page)

in human CTIP2KO neurons, Q175 mouse striata contained significantly higher levels of Ppp3cc than WT littermates (Figures 4C and 4D). However, no significant change was observed in the levels of Ppp3ca between WT and the two HD mouse models (Figures 4C and 4D). Moreover, an 80% decrease in pDARPP32-Thr34 levels was found in MSN cultures at 40 DIV derived from three independent HD human pluripotent stem cell (hPSC) lines compared with control cells (Figures 4E and 4F). Taken together, these results suggest that CTIP2 hypofunction in striatal MSNs may contribute to neuronal cell pathologies observed in HD.

DISCUSSION

We have developed an efficient and versatile platform for investigating the role of transcription factor CTIP2 in human MSN development and homeostasis. CTIP2-driven DEGs identified in the current study are significantly enriched in striatal genes associated with both direct and indirect pathways in the basal ganglia, which is in line with previous findings in mice (Arlotta et al., 2008). These *Ctip2*-deficient mice exhibited normal expression of immature MSN markers (*Meis2* and *Nolz1*) in the developing LGE at embryonic day 14.5. However, the number of Darpp32$^+$ neurons was significantly reduced in the newborn striatum. It remained unclear therefore whether Darpp32$^+$ MSNs could initially be generated in *Ctip2*$^{-/-}$ mice. Using a panel of LGE and postmitotic MSN markers, our study provides the first evidence that CTIP2 deficiency does not compromise the initial birth of MSNs from hESCs.

Moreover, this work demonstrates that CTIP2 plays a neuroprotective role against oxidative stress and is essential for maintaining striatal MSN characteristics. Notably, we reveal that CTIP2 deficiency causes an imbalance between protein kinase/phosphatase levels and activity, and subsequently reduced PKA target phosphorylation in MSNs, a cellular pathology shared by human and mouse MSNs carrying mHTT. Furthermore, our transcriptomic analysis highlights involvement of CTIP2 target genes in regulating Ca^{2+} signaling and kinase activity. *ADD3* and *CNGA3* regulate synaptic plasticity, Ca^{2+} transport, and activation of cAMP-dependent PKA, and were significantly downregulated in CTIP2-deficient MSNs (Bosia et al., 2016; Macneil et al., 1985). This provides mechanistic insight into how CTIP2 hypofunction may contribute to the reduced PKA-dependent protein phosphorylation via dysregulating Ca^{2+}-mediated cAMP activity in CTIP2-deficient MSNs, and potentially in HD (Figure 4G).

PKA and CDK5 phosphorylate DARPP32 and glutamate receptors, to modulate the excitability of striatal synapses and postsynaptic signaling in MSNs, in response to dopamine and glutamate stimulation (Bibb et al., 1999). Lower PKA activity has been associated with proteasome impairments in two HD mouse models and mHTT-expressing striatal cells (Lin et al., 2013). We demonstrate reduced phosphorylation levels of PKA targets DARPP32-Thr34 and GLUR1-Ser845 in both CTIP2KO and mHTT-expressing MSNs, potentially because of inhibited activation of PKA. Furthermore, we provide evidence that DARPP32 phosphorylation deficits can be directly rescued by restoring CTIP2 levels in KO MSNs.

When phosphorylated, pDARPP32-Thr34 acts as an inhibitor of PP1, which normally dephosphorylates PKA targets (Greengard et al., 1999). Intriguingly, PP1 also regulates phosphorylation of HTT at the Thr3 site and increases its aggregation properties (Branco-Santos et al., 2017). Thus, CTIP2 hypofunction-mediated disinhibition of PP1 activity due to reduced pDARPP32-Thr34 availability would induce mHTT aggregation in HD MSNs. A higher affinity for interaction between Ctip2 and mHtt than normal Htt was demonstrated in three independent HD

Figure 3. PKA-Dependent DARPP32-Thr34 and GLUR1-Ser845 Phosphorylation Is Regulated by CTIP2 in MSNs
(A) Within the PKA signaling gene set, CTIP2 target genes (*ADD3* and *CNGA3*) were significantly dysregulated and their change predicted to inhibit activation of cAMP-dependent PKA.
(B) Phosphorylation of PKA targets DARPP32-Thr34 and GLUR1-Ser845 is greatly reduced in CTIP2KO versus control MSNs.
(C) Quantification of (B) (left: n = 8, 17, 4; right: n = 10, 21, 4).
(D) Part of the PKA signaling pathway, levels of protein phosphatase 1 (PP1) and 3 (PP3) catalytic subunits are significantly increased in CTIP2KO versus control MSNs.
(E) Quantification of (D) (from left, top row: n = 9, 20, 4; n = 8, 16, 3; bottom row: n = 5, 12, 3; n = 5, 10, 3).
(F) Compared with control cells, CTIP2KO MSNs contain reduced levels of PKA catalytic subunits (PKAcat) and increased levels of CDK5R1, a neuron-specific activator of CDK5.
(G) Quantification of (F) (from left: n = 9, 22, 4; n = 8, 16, 3).
(H) Quantification of MAP2 levels shows no differences between control and CTIP2KO MSN cultures (n = 10, 22, 4).
(I) Reduced phosphorylation of DARPP32-Thr34 in CTIP2KO no. 4 MSNs is rescued by restoring CTIP2 levels (n = 6, 6, 3).
(C, E, and G–I) One-way ANOVA; $^*p < 0.05$, $^{**}p < 0.01$, $^{***}p < 0.001$, $^{****}p < 0.0001$; n.s., not significant. Data are presented as mean ± SEM for each genotype, with the means for individual clones indicated by red-shaded circles beside CTIP2KO data.
See also Figures S1E and S4.

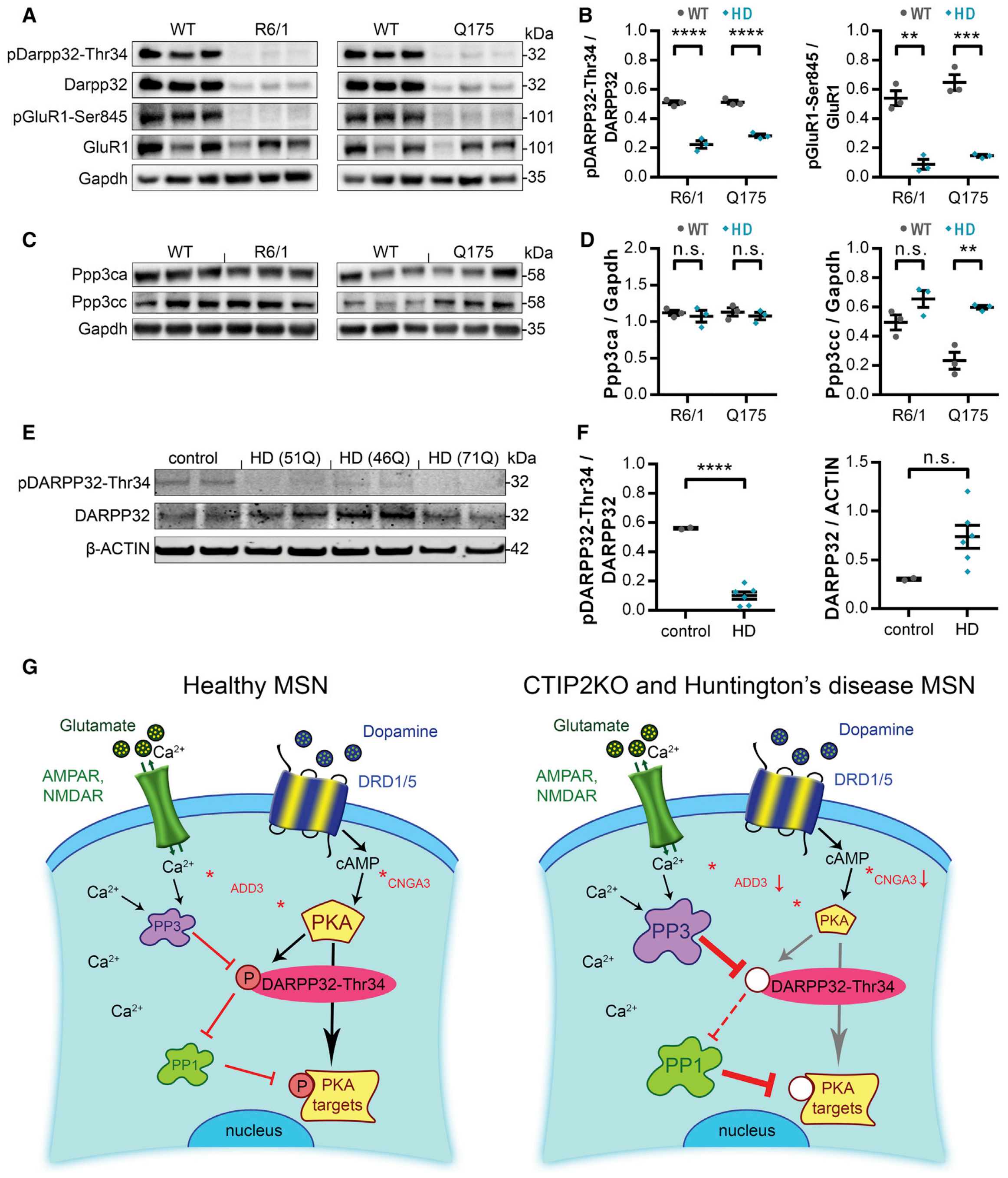

Figure 4. PKA-Dependent Protein Phosphorylation Deficits Are Shared by Human and Rodent mHTT-Expressing MSNs

(A and B) Images (A) and quantification (B) showing a significant mHtt-dependent decrease in pDarpp32-Thr34 and pGluR1-Ser845 levels in R6/1 and Q175 HD mouse models (B) (n = 3, 3).

(C and D) Images (C) and quantification (D) of two catalytic subunits of Pp3, Ppp3ca and Ppp3cc, showing a mHtt-dependent increase in Ppp3cc levels in R6/1 and Q175 mice (D) (n = 3, 3).

(E and F) Images (E) and quantification (F) showing a great mHtt-dependent decrease in pDARPP32-Thr34 levels in MSNs derived from three independent HD hPSC lines (F) (n = 2, 6, 2).

(B, D, and F) One-way ANOVA; $^{**}p < 0.01$, $^{***}p < 0.001$, $^{****}p < 0.0001$; n.s., not significant. Data are presented as mean ± SEM.

(G) Some of the CTIP2-regulated intracellular events are depicted in a healthy (left) versus CTIP2KO and HD (right) MSN. CTIP2 target genes in the PKA gene set are shown in red and some of their interactions with members of the PKA signaling pathway are indicated with an

(legend continued on next page)

mouse models, and mHtt was shown to impair transcriptional function of Ctip2 (Desplats et al., 2008). Thus, abnormal kinase/phosphatase activity in CTIP2-deficient and HD MSNs, in which mHTT interacts and disrupts CTIP2 function, may underlie not only disruption of synaptic signaling in the striatum but also mHTT aggregate formation, eventually leading to MSN degeneration and cell death.

The impact of this study may go beyond the HD field because genetic mutations associated with schizophrenia were found in genes enriched in MSNs (Skene et al., 2018). Reduced levels of full-length DARPP32 and increased levels of DARPP32 isoforms lacking the crucial residue Thr34 were reported in schizophrenia patients (Kunii et al., 2014), which would have major implications for MSN regulation by dopamine. Moreover, mutations in the *CTIP2* gene, causing either CTIP2 haploinsufficiency or a truncated CTIP2 protein, have been linked to a neurodevelopmental delay with speech impairment and intellectual disability in patients (Lessel et al., 2018).

In conclusion, we have explored CTIP2-regulated molecular mechanisms in striatal neurons and demonstrated an essential role for CTIP2 in human MSN homeostasis. This study provides a robust *in vitro* framework to study neurodevelopmental phenotypes and MSN dysfunction in the context of neurological disorders, with modulation of PKA-dependent protein phosphorylation representing a potential new therapeutic target.

EXPERIMENTAL PROCEDURES

CRISPR Design and Targeted Mutagenesis

Guide RNAs were synthesized as RNAs by *in vitro* transcription and transfected into HUES9 iCas9 hESCs as described by Gonzalez et al. (2014).

Cell Culture and MSN Differentiation

MSNs were differentiated from the following hPSC lines: HUES9 iCas9 and genome-edited derivatives (nos. 4, 33, and 34), HD hESCs (46Q and 51Q), and HD hiPSCs (71Q; see Supplemental Experimental Procedures for HD line details). MSNs were obtained and maintained as described previously (Arber et al., 2015).

Immunocytochemistry

Cells were incubated with primary antibodies overnight at 4°C followed by Alexa Fluor secondary antibodies for 1 h. Staining quantification was acquired manually in ImageJ (imagej.net) from >5,000 cells/sample blind to the experimental condition.

RNA-Seq

Paired-end sequencing was performed at Oxford Genomic Center on an Illumina HiSeq 4000 (Illumina, San Diego, USA).

Study Approval

Animal work was done under UK Home Office personal and project licenses in accordance with the requirements of the UK Animals (Scientific Procedures) Act 1986. Animals were group-housed and received water and food *ad libitum*.

Western Blot

Equal amounts of proteins for each sample were separated on 4%–12% Bolt Bis-Tris Plus gels (Thermo Fisher Scientific) and transferred via electro-blotting to a polyvinylidene difluoride membrane (0.45 μm pore size, GE Healthcare). Membranes were incubated with primary antibodies overnight at 4°C followed by secondary antibodies for 1 h. All blot images were quantified in ImageJ blind to the experimental condition.

Statistical Analysis

Statistical analysis was performed using one- or two-way ANOVA tests and considered statistically significant at $p < 0.05$. Sample sizes for each test are indicated in the figure legends as $n = n_1, n_2$, and n_3, where n_1 and n_2 are the number of control and CTIP2KO (or HD) samples, respectively, from n_3 independent experiments.

ACCESSION NUMBERS

RNA-seq data reported in this paper are available with the Sequence Read Archive under accession number SRA: SRP150394.

SUPPLEMENTAL INFORMATION

Supplemental Information can be found online at https://doi.org/10.1016/j.stemcr.2019.07.015.

AUTHOR CONTRIBUTIONS

M.F. and M.L. conceived the study and designed the experiments. M.F. carried out and analyzed the CTIP2 hESC and HD animal experiments with support from Z.L. and E.D. D.C.L.F. contributed to the RNA-seq data analysis and interpretation. M.L. (Paris) and A.L.P. contributed HD hPSC data and S.P.B. supplied HD animals. M.F. and M.L. wrote the manuscript and S.P.B. edited the manuscript.

ACKNOWLEDGMENTS

We thank Dr. Robert Andrews (Advanced Research Computing at Cardiff) for invaluable assistance with RNA-seq data analysis. This work was supported by EU Framework Program 7 *repair-HD* funding to M.L. and A.L.P., UK Medical Research Council grants to M.L., and EU Horizon 2020 JPND program *ModelPolyQ* funding to A.L.P.

asterisk. Decreased PKA signaling in CTIP2KO neurons is likely mediated through lower levels of catalytic subunits of PKA available and a loss of pDARPP32-Thr34-regulated inhibition of phosphatase PP1. Most of these molecular changes are also present in mHTT-expressing cells, suggesting that CTIP2 hypofunction might contribute to selective MSN pathology in HD.
See also Figure S4 and Table S4.

Received: December 11, 2018
Revised: July 25, 2019
Accepted: July 26, 2019
Published: August 22, 2019

REFERENCES

Arber, C., Precious, S.V., Cambray, S., Risner-Janiczek, J.R., Kelly, C., Noakes, Z., Fjodorova, M., Heuer, A., Ungless, M.A., Rodriguez, T.A., et al. (2015). Activin A directs striatal projection neuron differentiation of human pluripotent stem cells. Development *142*, 1375–1386.

Arlotta, P., Molyneaux, B.J., Jabaudon, D., Yoshida, Y., and Macklis, J.D. (2008). Ctip2 controls the differentiation of medium spiny neurons and the establishment of the cellular architecture of the striatum. J. Neurosci. *28*, 622–632.

Bibb, J.A., Snyder, G.L., Nishi, A., Yan, Z., Meijer, L., Fienberg, A.A., Tsai, L.H., Kwon, Y.T., Girault, J.A., Czernik, A.J., et al. (1999). Phosphorylation of DARPP-32 by Cdk5 modulates dopamine signalling in neurons. Nature *402*, 669–671.

Bosia, M., Pigoni, A., Zagato, L., Merlino, L., Casamassima, N., Lorenzi, C., Pirovano, A., Smeraldi, E., Manunta, P., and Cavallaro, R. (2016). ADDing a piece to the puzzle of cognition in schizophrenia. Eur. J. Med. Genet. *59*, 26–31.

Branco-Santos, J., Herrera, F., Pocas, G.M., Pires-Afonso, Y., Giorgini, F., Domingos, P.M., and Outeiro, T.F. (2017). Protein phosphatase 1 regulates huntingtin exon 1 aggregation and toxicity. Hum. Mol. Genet. *26*, 3763–3775.

Desplats, P.A., Lambert, J.R., and Thomas, E.A. (2008). Functional roles for the striatal-enriched transcription factor, Bcl11b, in the control of striatal gene expression and transcriptional dysregulation in Huntington's disease. Neurobiol. Dis. *31*, 298–308.

Gonzalez, F., Zhu, Z., Shi, Z.-D., Lelli, K., Verma, N., Li, Q.V., and Huangfu, D. (2014). An iCRISPR platform for rapid, multiplexable, and inducible genome editing in human pluripotent stem cells. Cell Stem Cell *15*, 215–226.

Greengard, P., Allen, P.B., and Nairn, A.C. (1999). Beyond the dopamine receptor: the DARPP-32/protein phosphatase-1 cascade. Neuron *23*, 435–447.

Hemmings, H.C., Greengard, P., Tung, H.Y.L., and Cohen, P. (1984). DARPP-32, a dopamine-regulated neuronal phosphoprotein, is a potent inhibitor of protein phosphatase-1. Nature *310*, 503–505.

Hodges, A., Strand, A.D., Aragaki, A.K., Kuhn, A., Sengstag, T., Hughes, G., Elliston, L.A., Hartog, C., Goldstein, D.R., Thu, D., et al. (2006). Regional and cellular gene expression changes in human Huntington's disease brain. Hum. Mol. Genet. *15*, 965–977.

Kunii, Y., Hyde, T.M., Ye, T., Li, C., Kolachana, B., Dickinson, D., Weinberger, D.R., Kleinman, J.E., and Lipska, B.K. (2014). Revisiting DARPP-32 in postmortem human brain: changes in schizophrenia and bipolar disorder and genetic associations with t-DARPP-32 expression. Mol. Psychiatry *19*, 192–199.

Langfelder, P., Cantle, J.P., Chatzopoulou, D., Wang, N., Gao, F.Y., Al-Ramahi, I., Lu, X.H., Ramos, E.M., El-Zein, K., Zhao, Y.N., et al. (2016). Integrated genomics and proteomics define huntingtin CAG length-dependent networks in mice. Nat. Neurosci. *19*, 623–633.

Lessel, D., Gehbauer, C., Bramswig, N.C., Schluth-Bolard, C., Venkataramanappa, S., van Gassen, K.L.I., Hempel, M., Haack, T.B., Baresic, A., Genetti, C.A., et al. (2018). BCL11B mutations in patients affected by a neurodevelopmental disorder with reduced type 2 innate lymphoid cells. Brain *141*, 2299–2311.

Lim, R.G., Salazar, L.L., Wilton, D.K., King, A.R., Stocksdale, J.T., Sharifabad, D., Lau, A.L., Stevens, B., Reidling, J.C., Winokur, S.T., et al. (2017). Developmental alterations in Huntington's disease neural cells and pharmacological rescue in cells and mice. Nat. Neurosci. *20*, 648–660.

Lin, J.-T., Chang, W.-C., Chen, H.-M., Lai, H.-L., Chen, C.-Y., Tao, M.-H., and Chern, Y. (2013). Regulation of feedback between protein kinase A and the proteasome system worsens Huntington's disease. Mol. Cell. Biol. *33*, 1073–1084.

Macneil, S., Lakey, T., and Tomlinson, S. (1985). Calmodulin regulation of adenylate-cyclase activity. Cell Calcium *6*, 213–226.

Nishi, A., Snyder, G.L., Nairn, A.C., and Greengard, P. (1999). Role of calcineurin and protein phosphatase-2A in the regulation of DARPP-32 dephosphorylation in neostriatal neurons. J. Neurochem. *72*, 2015–2021.

Onorati, M., Castiglioni, V., Biasci, D., Cesana, E., Menon, R., Vuono, R., Talpo, F., Goya, R.L., Lyons, P.A., Bulfamante, G.P., et al. (2014). Molecular and functional definition of the developing human striatum. Nat. Neurosci. *17*, 1804–1815.

Ring, K.L., An, M.C., Zhang, N., O'Brien, R.N., Ramos, E.M., Gao, F., Atwood, R., Bailus, B.J., Melov, S., Mooney, S.D., et al. (2015). Genomic analysis reveals disruption of striatal neuronal development and therapeutic targets in human Huntington's disease neural stem cells. Stem Cell Reports *5*, 1023–1038.

Simon, R., Baumann, L., Fischer, J., Seigfried, F.A., De Bruyckere, E., Liu, P., Jenkins, N.A., Copeland, N.G., Schwegler, H., and Britsch, S. (2016). Structure-function integrity of the adult hippocampus depends on the transcription factor Bcl11b/Ctip2. Genes Brain Behav. *15*, 405–419.

Skene, N.G., Bryois, J., Bakken, T.E., Breen, G., Crowley, J.J., Gaspar, H.A., Giusti-Rodriguez, P., Hodge, R.D., Miller, J.A., Muñoz-Manchado, A.B., et al. (2018). Genetic identification of brain cell types underlying schizophrenia. Nat. Genet. *50*, 825–833.

Tang, B., Di Lena, P., Schaffer, L., Head, S.R., Baldi, P., and Thomas, E.A. (2011). Genome-wide identification of Bcl11b gene targets reveals role in brain-derived neurotrophic factor signaling. PLoS One *6*, e23691.

Wei, T.T., Chen, C., Hou, J.W., Xin, W.J., and Mori, A. (2000). Nitric oxide induces oxidative stress and apoptosis in neuronal cells. Biochim. Biophys. Acta *1498*, 72–79.

Yger, M., and Girault, J.A. (2011). DARPP-32, jack of all trades... master of which? Front. Behav. Neurosci. *5*, 56.

Zhang, Z., Sun, T., Niu, J.G., He, Z.Q., Liu, Y., and Wang, F. (2015). Amentoflavone protects hippocampal neurons: anti-inflammatory, antioxidative, and antiapoptotic effects. Neural Regen. Res. *10*, 1125–1133.

Stem Cell Reports, Volume *13*

Supplemental Information

CTIP2-Regulated Reduction in PKA-Dependent DARPP32 Phosphorylation in Human Medium Spiny Neurons: Implications for Huntington Disease

Marija Fjodorova, Morgane Louessard, Zongze Li, Daniel C. De La Fuente, Emma Dyke, Simon P. Brooks, Anselme L. Perrier, and Meng Li

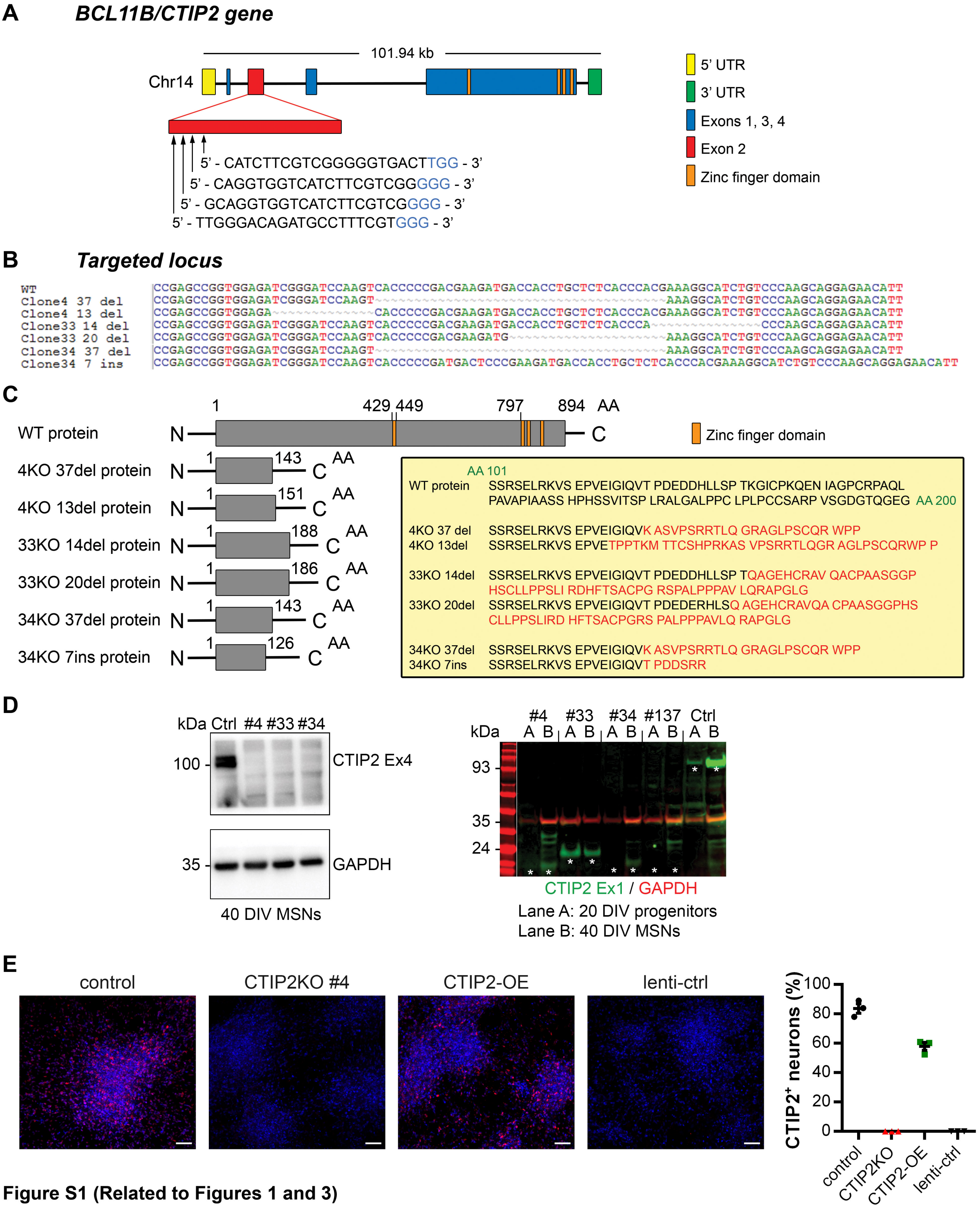

Figure S1 (Related to Figures 1 and 3)

Generation of CTIP2KO hESC lines with CRISPR/Cas9 assisted gene targeting technology. (A) Schematic illustration of the human CTIP2 gene and exon 2 targeting strategy with four gRNAs. **(B)** DNA sequences of the targeted locus in control and three independent CTIP2-/- lines (#4, #33 and #34) showing generated deletions/insertions. **(C)** Schematic illustrations and predicted amino acid sequences of truncated CTIP2 proteins resulting from frameshift mutations in CTIP2-/- lines. **(D)** Western blot analysis of CTIP2-/- lines confirms complete loss of the full-length CTIP2 protein (left) and presence of truncated CTIP2 protein isoforms (right) in CTIP2KO MSNs at 20 and 40 DIV. **(E)** Images and quantification of CTIP2^{+} cells in MSN cultures at 40DIV derived in following conditions: control, CTIP2KO #4 untreated, CTIP2KO #4 infected with either CTIP2-expressing or control lentivirus.

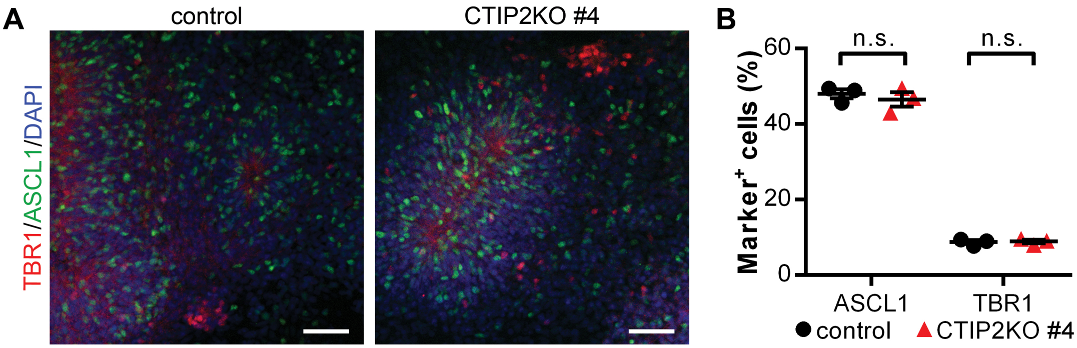

Figure S2 (Related to Figure 1)

Loss of CTIP2 does not affect regional specification of hESC-derived forebrain progenitors. Images (**A**) and quantification (**B**) of LGE-like progenitors in control and CTIP2KO #4 cultures at 20 DIV immunostained for subpallial marker ASCL1 and cortical marker TBR1. All marker+ cells were found not to differ significantly; one-way ANOVA. Mean ± s.e.m.; n.s., not significant. Scale bars: 50μm.

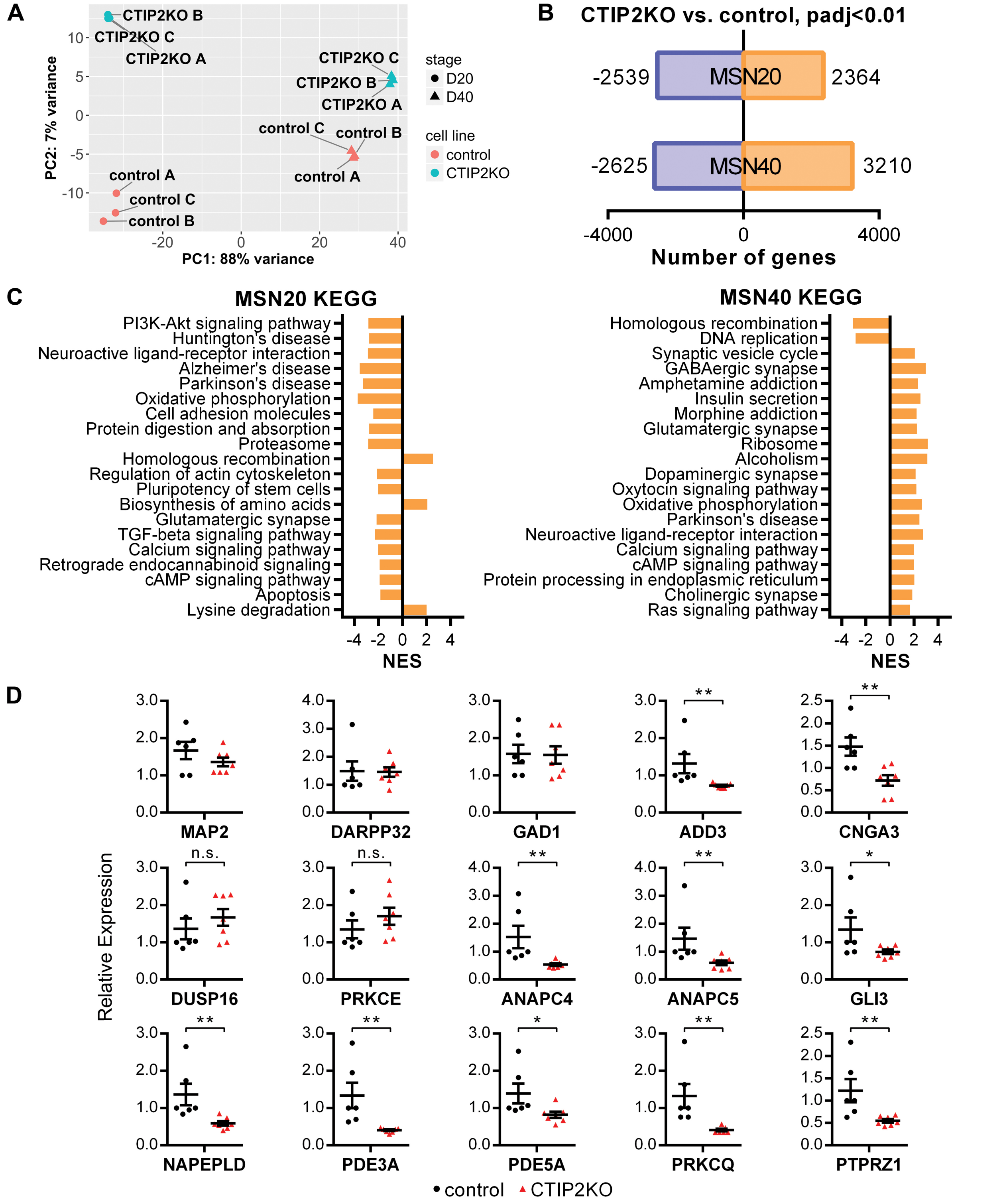

Figure S3 (Related to Figure 2)

RNA-seq analysis of D20 and D40 control and CTIP2KO MSNs with RT-PCR analysis of PKA pathway genes. (A) Principal component analysis of gene expression data for control and CTIP2KO #4 samples (n=3 biological replicates). **(B)** Numbers of DEGs at FDR(padj)<0.01 [upregulated genes shown in orange and downregulated genes in purple]. **(C)** KEGG pathway analysis of D20 and D40 DEGs. **(D)** RT-PCR analysis of PKA pathway genes in D40 control and CTIP2KO MSNs. One-way ANOVA or Mann-Whitney test; *,**p<0.05, 0.01; n.s., not significant [n=6,7,2].

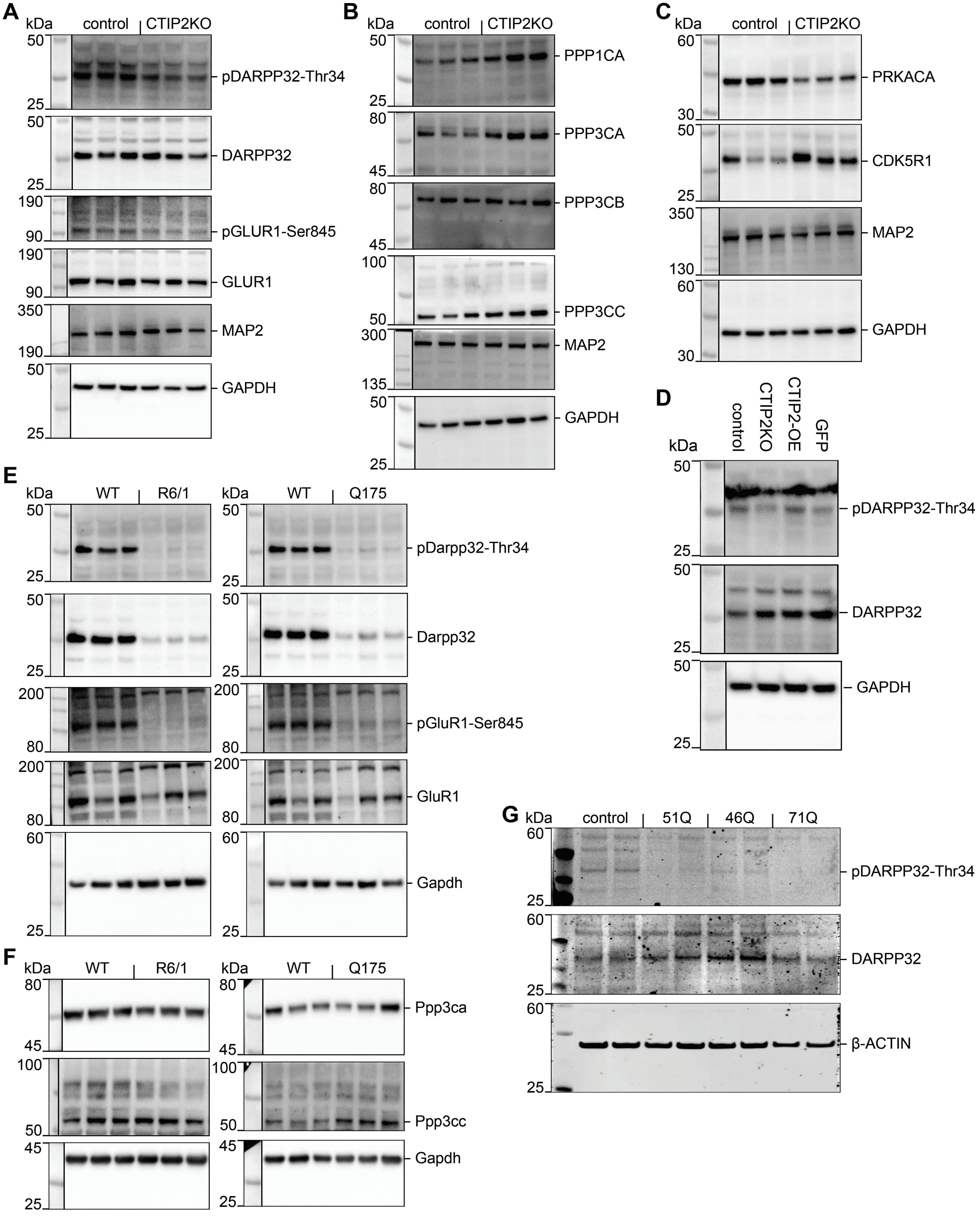

Figure S4 (Related to Figures 3 and 4)

Uncropped western blot images with markers. (A) Corresponding uncropped images of western blot shown in Figure 3B. **(B)** Corresponding uncropped images of western blot shown in Figure 3D. **(C)** Corresponding uncropped images of western blot shown in Figure 3F. **(D)** Corresponding uncropped images of western blot shown in Figure 3I. **(E)** Corresponding uncropped images of western blot shown in Figure 4A. **(F)** Corresponding uncropped images of western blot shown in Figure 4C. **(G)** Corresponding uncropped images of western blot shown in Figure 4E.

Supplemental Table Titles and Legends

Table S1 (Related to Figures 2 and S3) - Differentially expressed genes in CTIP2KO MSNs at 20 and 40 DIV. Gene lists are presented in a separate sheet for each time point (FDR<0.01). The first three columns identify the gene and the other columns contain differential expression statistics for CTIP2KO versus control group comparisons.

Table S2 (Related to Figures 2, 3 and S3) - Summary of striatal gene set, KEGG gene set and IPA canonical pathway enrichment analysis of CTIP2KO DEGs. The first sheet provides an overview and Fisher's exact test statistics for each association with striatal dataset tested. Tables #2-7 provide lists of striatum-specific MSN20 and MSN40 DEGs with differential expression statistics, each sheet corresponding to one 'time point'/'striatal dataset' combination. Tables #8-11 contain one sheet per 'time point'/analysis combination (KEGG or IPA) showing significantly dysregulated pathways (FDR<0.05), enrichment statistics and associated gene identifiers.

Table S3 (Related to Figures 2, 3 and S3) - Known CTIP2 target genes in CTIP2KO DEGs. This table contains one sheet per time point showing gene information and differential expression statistics.

Table S4 (Related to Figure 4) - Summary of HD dataset enrichment and functional annotation analysis of the CTIP2KO-HD DEGs. The first sheet provides an overview, Fisher's exact test statistics and names of dysregulated genes for each association tested. The following two sheets provide a compilation of MSN20 and MSN40 DEGs concordantly dysregulated (FDR<0.01) in at least one HD gene expression dataset. Each sheet corresponds to one time point and provides gene identifiers with differential expression statistics. Tables #4-5 contain one sheet per time point showing significantly dysregulated IPA canonical pathways (FDR<0.05), enrichment statistics and associated gene identifiers.

Supplemental Experimental Procedures

CRISPR Design and Targeted Mutagenesis

Guide RNAs (gRNAs) targeting the exon 2 of the *CTIP2* gene were selected using Zhang's lab online CRISPR Design tool (http://crispr.mit.edu/; **Figure S1A**). All gRNAs were synthesized as RNAs by *in vitro* transcription and transfected into iCas9 hESCs as described by Gonzalez *et al* (Gonzalez et al., 2014). Seven days post-transfection, individual colonies were isolated and clonally expanded. Genomic DNA was collected for each clone and the targeted region was screened by PCR (MyTaq, Bioline; forward primer: ACGCTCCGAGCTCAGGAAAG, reverse primer: GCAAGCGCAGCATCCCATAC; amplicon size: 142 bp). Genomic DNA samples showing insertions/deletions were then PCR amplified (forward primer: GTGGCCAGTGTCAAATGAAC, reverse primer: TCCTCACAGCAACCCTAATG; amplicon size: 706 bp) and cloned into a pGEM-T Easy vector (Promega) for Sanger sequencing (GATC Biotech). All studies were performed in three homozygous $CTIP2^{-/-}$ lines (#4, #33 and #34, referred to as CTIP2KO) and data is presented as the average between the three lines unless stated otherwise.

CTIP2 rescue

Transfer plasmid LV#1000-hPGK expressing human CTIP2 was a kind gift from Professor Malin Parmar (Lund). Production of lentivirus was performed in HEK293 cells using 2nd generation packaging system and following Lipofectamine 3000 kit guidelines (ThermoFisher Scientific). Virus was titrated in HEK cells using immunocytochemistry against CTIP2 and the resulting titer was calculated to be $9*10^9$ (U/mL). At 34 DIV, control and CTIP2KO MSNs were infected with the virus at a multiplicity of infection of 2 (MOI=number of lentiviral particles / number of target cells). At 40 DIV, cells were either fixed for immunocytochemistry or processed for protein extraction as described below.

Cell Culture and MSN Differentiation

All human pluripotent stem cell (hPSC) lines were maintained in feeder-free conditions on Matrigel-coated (Corning) plates in TeSR™-E8™ medium (STEMCELL Technologies) and passaged via manual dissociation using 0.02% EDTA pH7.2 (Merck Sigma-Aldrich). Medium spiny neurons (MSNs) were differentiated from the following hPSC lines: HUES9 iCas9 (Gonzalez et al., 2014) and genome edited derivatives (#4, #33, #34), SIVF018 [HD hESCs, 46Q (Bradley et al., 2011)], SI-187 [HD hESCs, 51Q (Verlinsky et al., 2005)], ND42228 [HD hiPSCs, 71Q, RRID:CVCL_1N96 from NHCDR]. MSNs were obtained and maintained as described previously (Arber et al., 2015). In short, neural differentiation was initiated by switching TeSR™-E8™ medium to DMEM-F12/Neurobasal (2:1; Thermo Fisher Scientific) supplemented with N2 (Thermo Fisher Scientific) and vitamin A-free B27 (Thermo Fisher Scientific) [referred to thereafter as N2B27] and this time point was noted as 0 days *in vitro* (0 DIV). For the first 10 DIV, cultures were supplemented with SB431542 (10 µM, Tocris), LDN-193189 (100 nM, StemGent) and dorsomorphin dihydrochloride (200 nM,Tocris). Splitting was done *en bloc* using EDTA at 9 DIV onto fibronectin-coated (Merck Millipore) plates at a ratio of 2:3. MSN fate was induced from 10 DIV by supplementing N2B27 medium with activin A (25 ng/ml, Cell Guidance Systems) until 30 DIV. Cultures were split again at 20 DIV onto poly-D-lysine hydrobromide (PDL, Merck Sigma-Aldrich) and laminin (Merck Sigma-Aldrich) co-coated plates at a density of 150k cells/cm^2. B27 with vitamin A (Thermo Fisher Scientific) was used from 20 DIV onwards. Differentiating MSNs were maintained in N2B27 supplemented with BDNF, GDNF (10ng/ml, PeproTech) from 25 DIV to aid neuronal maturation and survival.

Cytotoxicity Assay

MultiTox-Fluor Multiplex Cytotoxicity Assay (Promega) was performed following manufacturer's suggestions. Briefly, 96-well assay plates containing 40,000 MSN progenitors/well were set up at 20 DIV and cultures underwent terminal MSN differentiation. At 40 DIV, test compounds and vehicle controls were added to the cells. Treatment groups were as follows: untreated, SNAP (1000 µM) for 24 hours, amentoflavone (50 µM) for 24 hours, amentoflavone (50 µM) for 2 hours prior to addition of SNAP (1000 µM) for 24 hours (**Figure 1G**). The following controls were also included in each experiment: no cell control to determine background fluorescence, vehicle control, positive control for cytotoxicity [cells were treated with digitonin (Merck Sigma-Aldrich) at a final concentration of 50 µg/ml for 30 minutes]. Cytotoxicity assay reagents were added in an equal volume (100 µl/well) to all wells and incubated with samples for 30 minutes at 37°C. Fluorescence intensity was then measured on a microplate reader to assess viability (400Ex/505Em) and cell death (485Ex/520Em).

Immunocytochemistry

Cultured cells were rinsed in PBS and fixed in 3.7% PFA for 15 minutes. Cells were permeabilized in 0.3% Triton-X-100 solution in PBS (PBS-T) and then blocked in PBS-T containing 1% BSA and 3% donkey serum.

Cells were incubated with primary antibodies in blocking solution overnight at 4ºC. Following three PBS-T washes, Alexa-Fluor secondary antibodies (Thermo Fisher Scientific) were added at 1:1000 in blocking solution for 1 hour at ambient temperature in the dark. Cells were stained with DAPI at 1:1000 (Thermo Fisher Scientific). The following primary antibodies were used for the immunofluorescence studies: rabbit anti-GSH2 (Merck Millipore #ABN162, 1:500), goat anti-FOXP2 (Abcam #ab1307, 1:200), mouse anti-ISL1 (Developmental Studies Hybridoma Bank # 39.4D5, 1:500), goat anti-MEIS2 (Santa Cruz Biotechnology #sc-10599, 1:250), rabbit anti-FOXP2 (Abcam # ab16046, 1:500), mouse anti-FOXP1 (Abcam # ab32010, 1:800), rabbit anti-DARPP32 (Santa Cruz Biotechnology #sc-11365, 1:500), rabbit anti-TBR1 (Abcam # ab31940, 1:500), mouse anti-ASCL1 (BD Biosciences #556604, 1:500), rat anti-CTIP2 (Abcam # ab18465, 1:500). Images were taken on a Zeiss LSM710 confocal microscope from at least 5 randomly selected fields/sample and staining quantification was acquired manually in ImageJ (imagej.net) from >5,000 cells/sample blind to the experimental condition.

RNA Sequencing Data Analysis

Total RNA was extracted from TRIzol lysates using the PureLink RNA mini kit (Ambion) from three biological replicates per genotype at 20 and 40 DIV. These time points were chosen to reflect the onset of CTIP2 expression in nascent post mitotic MSNs and subsequent rapid increase in CTIP2 levels during differentiation, as well as to correspond to the time points at which several cellular pathologies have been observed. Paired-end sequencing was performed at Oxford Genomic Centre on an Illumina HiSeq 4000 (Illumina, San Diego, USA) obtaining a library size of ~80 million reads per sample. FASTQ files were trimmed and mapped to the Ensembl human genome GRCh38.84 (hg38) using STAR (v2.5.1b). Quality of the samples was assessed using FastQC (v0.11.2) prior to and after trimming. Gene counts were obtained from the number of uniquely aligned unambiguous reads by Subread:featureCount (v1.4.6-p2). Library size normalization and differentially expressed genes (DEGs) were determined using the R/Bioconductor package DESeq2 (v1.14.1) (Love et al., 2014). Benjamini-Hochberg (BH) test for multiple correction was used to control the false discovery rate (FDR). Subsequent study of KEGG gene set and pathway enrichment analysis was performed on protein coding DEGs with an EntrezID and FDR<0.01 using the Ingenuity Pathway Analysis (Qiagen) and R/Bioconductor package clusterProfiler (v3.12.14) (Yu et al., 2012). The associations between present CTIP2KO gene sets and published striatal and HD gene sets were determined by Fisher's exact test, performed in RStudio (www.r-project.org).

PCR Analysis

Total mRNA was extracted from TRIzol lysates using the PureLink RNA mini kit (Ambion) from three biological replicates per line at 40 DIV. RT-PCR was completed using 2 ug mRNA and the EvoScript kit (Roche). MesaGreen kit was used to perform qPCR using 200 pg mRNA/reaction to validate CTIP2KO DEGs in the PKA gene set. The following primers were used:

Gene Name	Forward Primer	Reverse Primer
MAP2	CAACGGAGAGCTGACCTCA	CTACAGCCTCAGCAGTGACTA
DARPP32	GGTATTTTTATCCGTGCGCGAAC	CTTCCTCCTCTGGTGAGGAGTG
GAD1	CGAGGACTCTGGACAGTAGAGG	GATCTTGAGCCCCAGTTTTCTG
ADD3	CAGCCAAGGCGTGATTACCA	TCTTCCCGAAAGGCAGGACT
CNGA3	GGGACCGGACTCTTTTCCTG	CACCACGATCGCATCCTTCT
DUSP16	ACGATCAAAGCTCCCAAGATGT	GCCAGGGAAACAACGAGAGA
PRKCE	CAACGGCCACAAGTTCATGG	AGGTGCAGACTTGACACTGG
ANAPC4	ATTTTCCTGGTCTGGTCGCC	CCAGACACGTCACCTCCTTT
ANAPC5	GGTGCTGCTGAACGAGATGAG	GAATTTGCCAGCTGTGGACAAG
GLI3	GCCTCCAGCACCACTTCTAAT	TCAATGAGGCCCTCTCGTCA
NAPEPLD	TCGGAGCTTATGAACCGAGG	AGGCAAAAGTTCCCCAGTGA
PDE3A	ACAGGTCTACCCACCTTGGA	AGGATCTGCTTTTGGTGAGGG
PDE5A	TGGTGAGCCCTTGAACATCA	CTGGGCTACACCAACAACCT
PRKCQ	AAAGGTCCACCACGTCAAGT	GTAGCCCTGTTTGTTCAGGC
PTPRZ1	TTGTTGAAGAGATTGGCTGGTC	TTGATAGGAGATTGTTTTGGGCT

Study Approval

All animal work was done under UK Home Office personal and project licenses in accordance with the requirements of the UK Animals (Scientific Procedures) Act 1986 and European Directive 2010/63/EU and with the approval of the local Cardiff University Ethics Review Committee. Animals were group-housed and received water and food *ad libitum*.

Western Blot Analysis

At 40 DIV, cells were collected in PBS and lysed on ice in RIPA buffer (Abcam) supplemented with protease and phosphatase inhibitors (Merck Sigma-Aldrich). Striatal region was dissected out from adult heterozygous HD mice and respective WT littermates (R6/1: 5 months, Q175: 24 months). Tissues were triturated by pipetting and lysed as described above. Protein concentrations of whole cell lysates were measured using the DC protein assay kit (BioRad) and then combined with 1X Bolt LDS Sample Buffer (Thermo Fisher Scientific) and 1X Bolt Sample Reducing Agent (Thermo Fisher Scientific). Equal amounts of proteins for each sample were separated on 4-12% Bolt Bis-Tris Plus gels (Thermo Fisher Scientific) and transferred via electro-blotting to a PVDF membrane (0.45 μm pore size, GE Healthcare). Membranes were blocked with 5% BSA in TBS containing 0.1% Tween-20 (TBS-T) for 2 hours at ambient temperature and then incubated with primary antibodies overnight at 4°C. The following primary antibodies were used for the western blot studies: rabbit anti-pDARPP32-Thr34 (Cell Signaling Technology #12438-D27A4, 1:1000), rabbit anti-DARPP32 (Santa Cruz Biotechnology #sc-11365, 1:500), rabbit anti-pGLUR1-Ser845 (Cell Signaling Technology #8084-D10G5, 1:1000), rabbit anti-GLUR1 (Cell Signaling Technology #13185-D4N9V, 1:1000), mouse anti-PP1α (PPP1CA, Santa Cruz Biotechnology #sc-271762, 1:1000), mouse anti-PP2B-Aα (PPP3CA, Santa Cruz Biotechnology #sc-17808, 1:1000), mouse anti-PP2B-Aβ (PPP3CB, Santa Cruz Biotechnology #sc-365612, 1:1000), mouse anti-PP2B-Aγ (PPP3CC, Santa Cruz Biotechnology #sc-293361, 1:1000), rabbit anti-PKA catalytic subunit (PKAcat, Abcam #ab76238, 1:5000), rabbit anti-p35/25 (CDK5R1, Cell Signaling Technology #2680-C64B10, 1:1000), rabbit anti-MAP2 (Merck Sigma-Aldrich #AB5622, 1:1000), rabbit anti-CTIP2 (Abcam #ab70453, 1:2000), rat anti-CTIP2 (Abcam # ab18465, 1:1000), mouse anti-GAPDH (Abcam #8245, 1:10000), mouse anti-β-ACTIN (Merck Sigma-Aldrich #A3854, 1:50000). Following three TBS-T washes, membranes were incubated with a horseradish peroxidase-conjugated anti-mouse or anti-rabbit antibody (Abcam) for 1 hour at ambient temperature. For CTIP2/GAPDH blotting (**Figure S1D**), membranes were incubated with anti-rat IRDye 800CW and anti-mouse IRDye 680RD secondary antibodies (LI-COR). Blots were developed with ECL system (Thermo Fisher Scientific) and imaged on an Odyssey CLx imaging system (LI-COR) or on a Gel Doc XR system (Bio-Rad). All images were quantified in ImageJ blind to the experimental condition.

Statistical Analysis

RNA sequencing data was analyzed separately as described above. SPSS Statistics 20 software (IBM) was used for all other statistical analyses. All quantified data were plotted in Prism 6 (GraphPad Software) and are reported as mean ± SEM with sample sizes for each test indicated in the figure legends. Horizontal line on dot plots depicts mean ± SEM for each genotype, with the means for individual clones indicated by red-shaded circles beside CTIP2KO data. Normal distribution of data was assessed the Shapiro-Wilk test. Statistical analysis was performed using one-/two-way ANOVA tests followed by Bonferroni's correction for multiple comparisons if applicable or non-parametric Mann-Whitney test for not normally distributed data. Results were considered statistically significant at $p<0.05$. No statistical methods were used to predetermine sample sizes, but our sample sizes are similar to those reported in previous publications (Arber et al., 2015; Cavey et al., 2016; Victor et al., 2018). Randomization was used to assign experimental conditions and collect data, and data collection was always done in parallel with controls. Data analyses were not performed blind to the conditions of the experiments except where stated otherwise.

Data and Software Availability

RNA-seq data reported in this paper are available with the SRA accession number **SRP150394** (https://www.ncbi.nlm.nih.gov/sra/SRP150394). No custom algorithms were used to analyze data in this study. The authors declare that all data supporting the findings of this study are available within this article, its Supplemental Information files, or are available from the corresponding authors upon reasonable request.

Supplemental References

Arber, C., Precious, S.V., Cambray, S., Risner-Janiczek, J.R., Kelly, C., Noakes, Z., Fjodorova, M., Heuer, A., Ungless, M.A., Rodriguez, T.A., *et al.* (2015). Activin A directs striatal projection neuron differentiation of human pluripotent stem cells. Development *142*, 1375-1386.

Cavey, M., Collins, B., Bertet, C., and Blau, J. (2016). Circadian rhythms in neuronal activity propagate through output circuits. Nat. Neurosci. *19*, 587-595.

Gonzalez, F., Zhu, Z., Shi, Z.-D., Lelli, K., Verma, N., Li, Q.V., and Huangfu, D. (2014). An iCRISPR Platform for Rapid, Multiplexable, and Inducible Genome Editing in Human Pluripotent Stem Cells. Cell Stem Cell *15*, 215-226.

Love, M.I., Huber, W., and Anders, S. (2014). Moderated estimation of fold change and dispersion for RNA-seq data with DESeq2. Genome Biol. *15*, 550.

Victor, M.B., Richner, M., Olsen, H.E., Lee, S.W., Monteys, A.M., Ma, C., Huh, C.J., Zhang, B., Davidson, B.L., Yang, X.W., *et al.* (2018). Striatal neurons directly converted from Huntington's disease patient fibroblasts recapitulate age-associated disease phenotypes. Nat. Neurosci. *21*, 341-352.
Yu, G., Wang, L.-G., Han, Y., and He, Q.-Y. (2012). clusterProfiler: an R Package for Comparing Biological Themes Among Gene Clusters. OMICS *16*, 284-287.
